# Out‐of‐field neutron radiation from clinical proton, helium, carbon, and oxygen ion beams

**DOI:** 10.1002/mp.17797

**Published:** 2025-04-04

**Authors:** Matteo Bolzonella, Marco Caresana, Andrea Cirillo, Josep M. Martí‐Climent, Evangelina Martínez‐Francés, Christina Mooshammer, Stefan Schmidt, Stephan Brons, Marco Silari, Christina Stengl, Liliana Stolarczyk, José Vedelago

**Affiliations:** ^1^ Department of Energy Politecnico di Milano Milan Italy; ^2^ Medical Physics and Radiation Protection Department Clínica Universidad de Navarra Pamplona Spain; ^3^ Medical Physics and Radiation Protection Department Clínica Universidad de Navarra Madrid Spain; ^4^ Department of Medical Physics in Radiation Oncology German Cancer Research Center (DKFZ) Heidelberg Germany; ^5^ Heidelberg Institute for Radiation Oncology (HIRO) National Center for Radiation Research in Oncology (NCRO) Heidelberg Germany; ^6^ Department of Physics and Astronomy Heidelberg University Heidelberg Germany; ^7^ Department of Radiation Oncology Heidelberg University Hospital (UKHD) Heidelberg Germany; ^8^ Medical Faculty Heidelberg Heidelberg University Heidelberg Germany; ^9^ Heidelberg Ion Beam Therapy Center (HIT) Heidelberg University Hospital Heidelberg Germany; ^10^ CERN Geneva 23 Switzerland; ^11^ Danish Centre for Particle Therapy Aarhus University Hospital Aarhus Denmark

## Abstract

**Background:**

In hadron therapy, out‐of‐field doses, which may in the long‐term cause secondary cancers, are mostly due to neutrons. Very recently, ^4^He and ^16^O beams have been added to protons and ^12^C ions for clinical therapy.

**Purpose:**

The focus of this article is to compare secondary neutron doses produced by clinical protons, ^4^He, ^12^C, and ^16^O ion beams.

**Methods:**

Ambient dose equivalent, H*(10), measurements were performed, with five types of rem counters, of the neutron field produced by the four primary ions impinging on a water phantom. This experiment was performed at the Heidelberg Ion Beam Therapy Center (HIT) in the framework of the activities of the European Radiation Dosimetry Group (EURADOS). The experimental data are normalized to both unit primary particle and target dose, and are further compared to Monte Carlo (MC) simulations performed with the FLUKA and MCNP codes.

**Results:**

The intensity of the neutron field increases with ion mass, and the trend is more significant in the forward direction. The minimum H*(10) for all ions, 5µSv/Gy to 10µSv/Gy, was measured in the transverse and backward directions, whereas the maximum measured value was about 1.3 mSv/Gy for primary ^16^O ions in the forward direction. Additional MC simulations are presented for a more detailed analysis of the rem counters’ response in the presence of heavy charged fragments. In the downstream direction, for ^12^C and ^16^O ions, approximately only 30% of the instruments’ counts are due to neutrons.

**Conclusion:**

The four extended‐range instruments provide reliable and consistent results, whereas the conventional rem counter underestimates H*(10) in a neutron field with a large high‐energy component. FLUKA and MCNP provide consistent predictions, within a factor of 1.6 for the downstream position and lower differences in the other cases, and are in agreement with the experimental data. It was found that under certain conditions neutrons do not represent the only secondary radiation field to be monitored; the presence of charged particles affects the performance of moderator‐type neutron detectors.

## 1 INTRODUCTION

After a long pioneering phase carried out at research laboratories, 35 years ago proton and light ion therapy, also known as hadron therapy, entered the clinical phase with the first hospital‐based proton therapy facilities.^1^ Proton therapy is now a well‐established treatment modality delivered in more than 100 hospitals worldwide, with more than 300 000 patients treated to date.^2^ Apart from early experimental treatments with various types of ions from helium to argon performed at the Berkely National Laboratory (BNL) in the USA, radiation therapy with ^12^C ion beams has gained momentum only in the past 10 years, although at a reduced scale compared to protons. This lack of momentum gain is caused by the larger size and cost of the accelerator and associated equipment requiring higher energies to treat deep‐seated tumors, the higher magnetic rigidity of carbon ions, and by the complexity, size, and cost of isocentric gantries.^3^ About 15 facilities are in clinical operation with approximately 50 000 patients treated to date.^2^

Hadron therapy is now entering a new phase, with clinical facilities considering or already using ^4^He and ^16^O beams for patient treatment. In Europe, these are the National Centre for Oncological Hadrontherapy (CNAO) in Italy,^4^ the Heidelberg Ion beam Therapy Center (HIT) in Germany,^5^ and MedAustron in Austria.^6^ The HIT facility was designed to deliver proton, helium, carbon, and oxygen beams with a fully active scanning system.^7^, ^8^, ^9^ Patients have been treated with protons and carbon ions for many years, while the first‐ever patient treatment with a scanning helium ion beam occurred in 2021.^10^, ^11^, ^12^ Although the technology for treatments with oxygen ions is available at HIT, it has not yet been used. However, the interest remains in view of potential benefits like increased relative biological effectiveness (RBE) and differences in neutron radiation compared to lighter ions.^13^, ^14^, ^15^

The evaluation of risk for second malignancies after radiation therapy is a long‐standing issue, which remains as the most advanced treatment modalities are introduced. In hadron therapy, out‐of‐field doses are mostly due to neutrons^16^, ^17^, ^18^, ^19^, ^20^: internal neutrons produced within the patient, and external neutrons generated within the beam delivery system and inside the treatment room. While the production of internal neutrons depends on the treatment plan, the production of external neutrons is facility dependent, and it is well known that this component is much larger with passive beam delivery systems than with beam scanning.^21^, ^22^, ^23^ Neutron production depends on primary ion, beam intensity, and energy per nucleon, with the energy distribution extending up to the maximum beam energy.^24^

For several years, the European Radiation Dosimetry group (EURADOS)^25^ has been investigating the production of secondary radiation (both in‐phantom and in‐room) from proton beams.^26^, ^27^, ^28^, ^29^, ^30^, ^31^, ^32^, ^33^, ^34^, ^35^ The last body of measurements by this group—of which this article addresses some of the results—was performed at HIT in November 2023. Both the secondary radiation dose to the patient, in water and anthropomorphic phantoms, was assessed with various types of passive dosimeters, and the scattered neutron component in the room (using a water phantom) was measured to provide information on neutron spectral distributions and neutron ambient dose equivalent, H*(10).

The instrument usually employed for measuring H*(10), the operational radiation protection quantity to which area monitors and survey meters are calibrated, is the rem counter.^36^ The response function of rem counters approximates the fluence to ambient dose equivalent conversion coefficients,^37^ h*(10), over a broad energy range. Conventional rem counters measure H*(10) from thermal up to 10 MeV to 15 MeV neutrons. Extended‐range rem counters measure H*(10) up to the GeV energy range.

This article reports on results of H*(10) measurements of the neutron field produced by clinical proton (also indicated with p in this article), ^4^He, ^12^C, and ^16^O ion beams. This experiment was performed in the framework of Working Groups (WG) 9 and 11 of EURADOS. The article discusses in‐room neutron H*(10) values measured with various types of rem counters, with focus on comparing the secondary neutron dose for the same target dose delivered by the four different primary ions. Monte Carlo (MC) simulations with the FLUKA and MCNP codes were performed in a simplified geometry to compare with the measured values. Additional MC simulations were carried out for a more detailed analysis of the rem counters’ response in the presence of heavy charged fragments.

## 2 MATERIALS AND METHODS

### 2.1 Experimental set‐up

The measurements were performed in the experimental room of HIT with dimensions 6.5 m × 8.0 m × 3.5 m, where an intensity‐modulated raster scanning technique is used for beam delivery.^7^, ^8^ The four primary ions available at HIT were used, namely protons, helium, carbon, and oxygen. The isocentre, located 145cm from the centre of the nozzle beam exit window at a height of 129.5cm above the floor, was used as a reference point for the positioning of the phantom and the detectors. A water phantom with dimensions of 60cm × 30cm × 30cm was used as in the work by Stolarczyk et al.^28^ The phantom has 15 mm thick polymethylmethacrylate (PMMA) walls and a 12cm × 12cm entrance window with a thickness of 4 mm PMMA. The position of the phantom was set such that the center of the entrance window was aligned with the isocentre of the room, with the phantom entrance surface placed at 130cm from the beam exit window.

**FIGURE 1:**
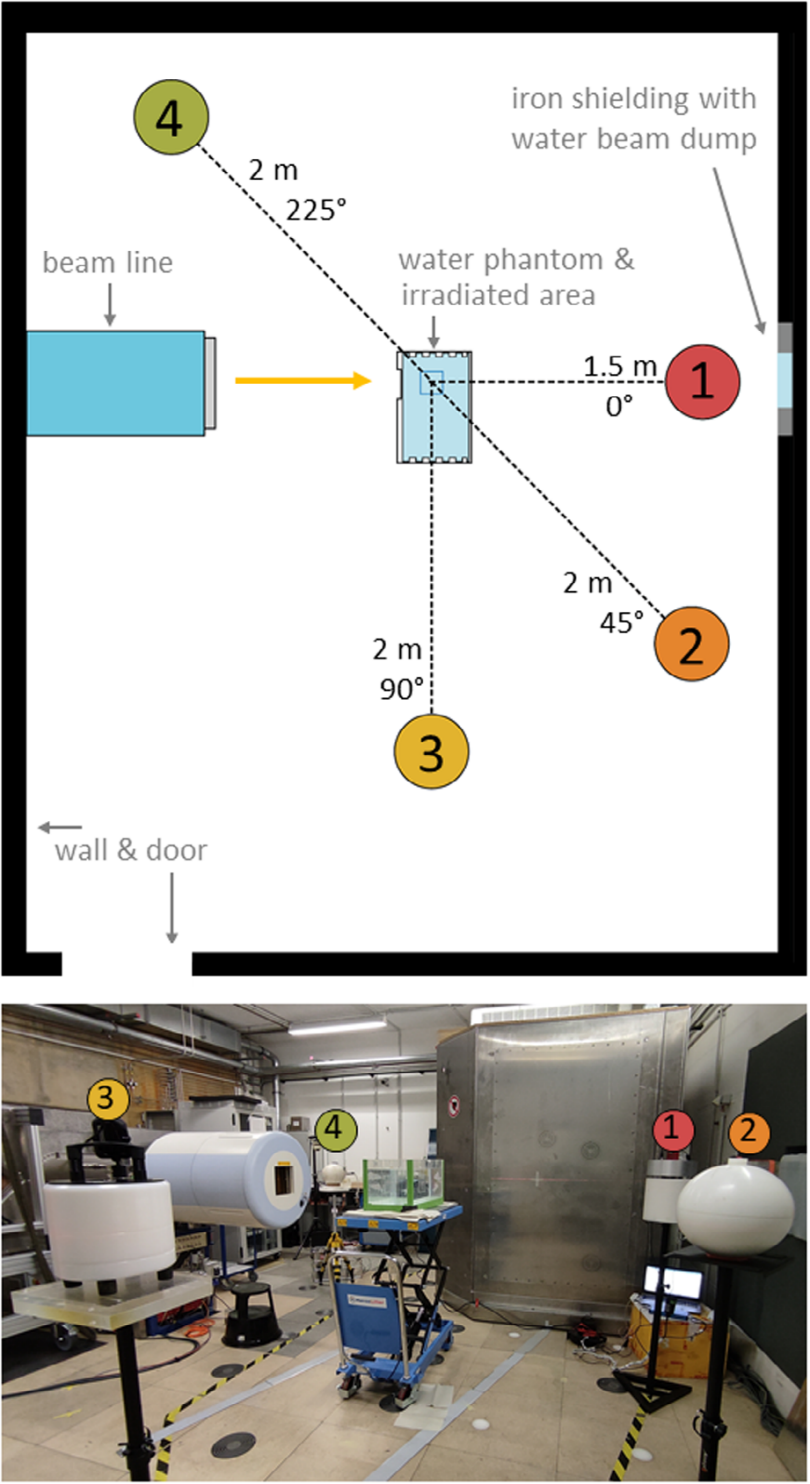
Top: sketch of the experimental room with the location of the water phantom and of the four measurement positions. Bottom: photograph showing the phantom placed downstream of the nozzle, with four rem counters in the measurement positions: LUPIN in Position 1, passive LINUS in Position 2, WENDI II in Position 3, and LINUS in Position 4 (in the background).

The rem counters were in turn placed in four locations: at 0° (Position 1), 45° (Position 2), 90° (Position 3), and 225° (Position 4) relative to the incoming beam direction (Figure 1). Position 1 was located at 150cm distance from the isocentre, whereas Positions 2, 3, and 4 were located at 200cm from the isocentre. The center of each of the rem counters was placed at the isocentre height, and the effective point of measurement was at the center of the device. The four positions were selected due to the constrained space in the experimental room and to imitate the positions used in a previous study.^26^, ^34^

To imitate a clinical beam, a spread‐out Bragg peak (SOBP) was delivered ranging from 10cm to 20cm depth in water, using a field size of 10cm × 10cm for the four ions. This irradiation geometry resulted in a cubic target with a volume of 10cm × 10cm × 10cm, already used in other facilities for similar experiments.^26^, ^28^, ^34^ Each SOBP plan was designed to deliver a physical dose between 1 Gy and 10 Gy to the target. The energy range, spot size, and number of delivered particles are given in Table 1.

**TABLE 1:** Ions, energy range, spot size range and number of particles used in the experiment. The number of particles is the total number of ions needed to deliver 1 Gy at the target volume.

Particle	Energy range (MeV/u)	Spot size range (mm)	Number of particles
p	118.75–171.60	9.9–13.5	7.20 × 10^10^
^4^He	119.78–172.77	9.0–10.8	1.97 × 10^10^
^12^C	227.29–331.47	9.9–10.3	4.00 × 10^9^
^16^O	268.64–396.17	10.1–10.5	2.64 × 10^9^

### 2.2 Rem counters

**FIGURE 2:**
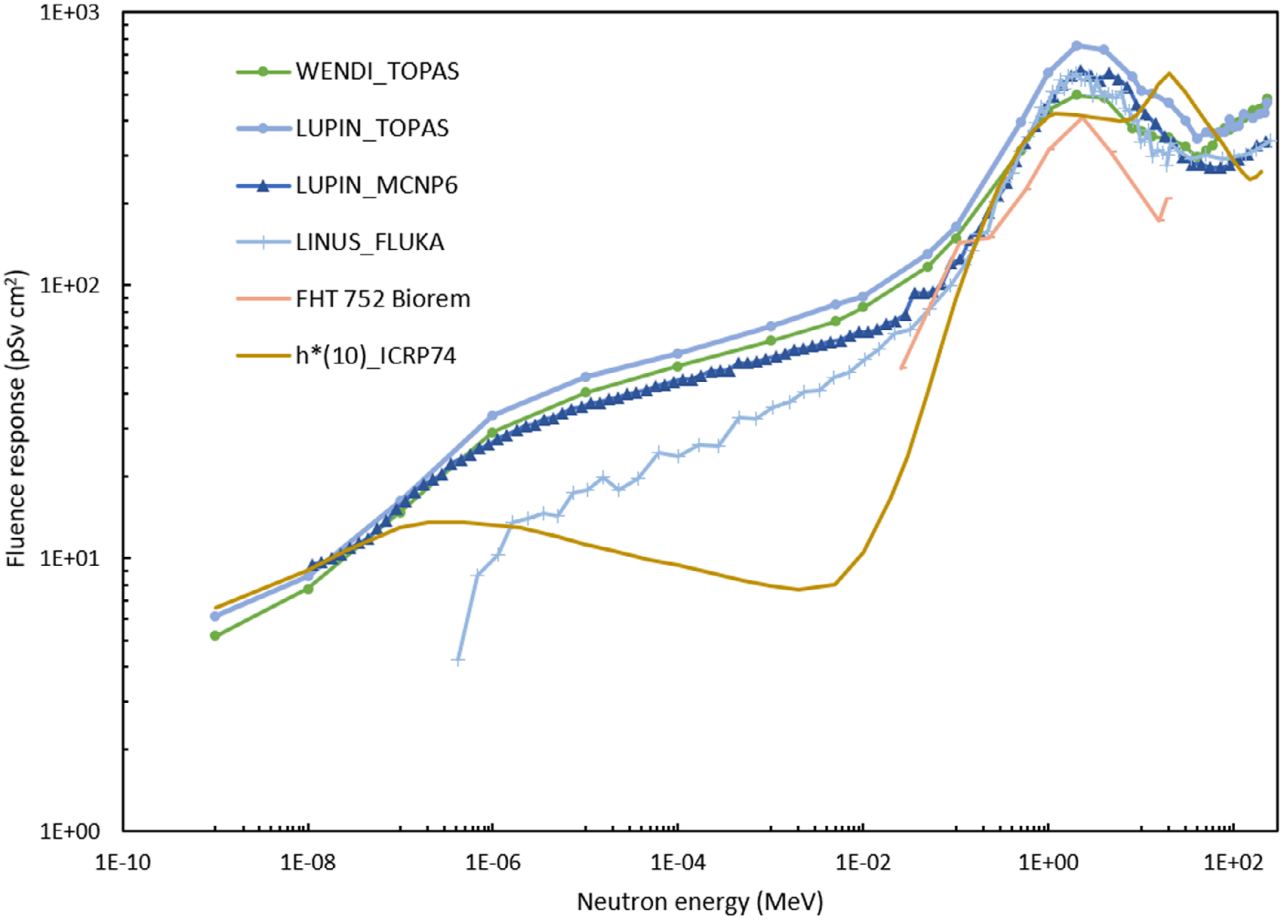
Absolute fluence responses of the neutron rem counters plotted along with the h*(10) coefficients from ICRP 74^37^. The response of the passive LINUS is not shown as it is analogous to the one of the LINUS. The response of the LUPIN was calculated with two different MC codes. The references for the various instruments are given in the text. The response of the BIOREM is the one provided by the manufacturer.

Five different types of rem counters were employed for the measurements, four with extended response (LINUS from CERN, LUPIN, and the passive LINUS from the Politecnico di Milano, WENDI II from the Clínica Universidad de Navarra) and a conventional one (BIOREM from DKFZ). Extended‐range rem counters incorporate a shell of high‐Z material in the polyethylene moderator, which extends the detector response to high‐energy neutrons (up to the GeV region) exploiting the generation of secondary neutrons via (n, xn) inelastic scattering reactions at neutron energies above 8 MeV. The response functions of all instruments are plotted in Figure 2. Their calibration factors and related uncertainties are given in Table 2. The quoted uncertainties for each rem counter include the AmBe calibration source output used for its calibration, the positioning of the instrument at the calibration point, and the calibration measurement uncertainty. Each instrument is briefly described below.

**TABLE 2:** Rem counters’ calibration factors and uncertainties.

Instrument	Calibration factor	Uncertainty (1σ) (%)	Calibration laboratory
LINUS	0.94 nSv/count	3.9	CERN^a^
LUPIN	0.60 nSv/count	4.0	CERN^a^
Passive LINUS	64 nSv/(tracks cm^2^)	6.5	CERN^a^
WENDI II	0.31 nSv/count	2.5	LPN CIEMAT^b^
BIOREM	0.55 nSv/count	4.8	PTB^c^

All instruments were calibrated with AmBe neutron sources.

^a^ European Organization for Nuclear Research, Geneva, Switzerland.

^b^ Laboratorio de Patrones neutrónicos, Centro de Investigaciones Energéticas, Medioambientales y Tecnológicas, Madrid, Spain.

^c^ Physikalisch‐Technische Bundesanstalt, Braunschweig, Germany.

#### 2.2.1 LINUS

The LINUS (Long Interval NeUtron Survey meter)^38^, ^39^, ^40^, ^41^ is the original extended‐range neutron rem counter developed about 35 years ago from an Andersson‐Braun type device. As compared to all past measurements, its electronics have been improved. The ^3^He counter is now connected to a PDT20A‐SHV‐5 V electronics module^42^ that provides the bias voltage and performs the signal processing. The bias voltage was set to 1200 V and the low‐level discriminator (LLD) to its maximum value to reject noise and events coming from interacting photons. The PDT20A was then connected to a LabJack T7Pro fast counter interface to record the generated pulses.

#### 2.2.2 LUPIN

The LUPIN (Long Interval, Ultra‐wide dynamic, Pile‐up free, Neutron rem counter)^43^, ^44^ uses front‐end electronics based on a logarithmic amplifier. The working principle is rather simple: the current generated inside the proportional counter is amplified with a current to voltage log‐amplifier and the output signal is acquired and digitalized with a Field Programmable Gate Arrays (FPGA). When the derivative of the acquired current exceeds a user‐settable threshold the instrument records a neutron interaction. In this way, it is possible to count the neutron events while effectively discriminating the signal induced by a steady photon field.

#### 2.2.3 Passive LINUS

The passive LINUS^45^, ^46^, ^47^ is the passive version of the LINUS. The instrument hosts solid‐state nuclear track detectors made of poly allyl diglycol carbonate (PADC), coupled to converters enriched in ^10^B. For this experiment, the converter consisted of B_4_C (99 % ^10^B) deposited on a thin metallic substrate, coupled to a single PADC detector. Thermal neutrons are detected via the ^10^B(n,α)^7^Li reaction. The α particle and the ^7^Li ion generate tracks in the PADC, which after etching become visible with an optical microscope. They can thus be distinguished from tracks due to naturally occurring radioactive material (NORM) and from plastic defects and/or dust particles mimicking tracks. The PADCs are read with the *Politrack* automatic system.^48^ The rem counter sensitivity is 15.7 × ·10^3^ tracks cm^−2^ mSv^−1^ which, when compared with a background track density of (45 ± 21) tracks cm^−2^, gives a lower detection limit in the order of 1 µSv. This system is insensitive to the gamma background as the PADC detector is intrinsically insensitive to photons.

#### 2.2.4 WENDI II

The WENDI II (Wide Energy Neutron Detection Instrument) is an extended‐range rem counter developed by Olsher et al.^49^ The upper side of the detector cavity is protected by a small boron doped rubber shield to prevent it from an over response due to low energy neutrons impinging on the top surface. Its absolute response in terms of pSv per cm^2^ was simulated in a previous work^50^ through TOPAS,^51^ a toolkit based on Geant4.

#### 2.2.5 FHT 752 BIOREM

The FHT 752 BIOREM is a commercial neutron dose rate monitor for both stationary and portable use, particularly suitable for environmental measurements.^49^, ^52^ Unlike all other instruments used in this experiment, it is a non‐extended‐range rem counter. For the thermal region, overestimation of the neutron dose needs to be considered, whereas for fast neutrons with energies above 10 MeV the device largely underestimates H*(10).

The main technical features of the five instruments are listed in Table 3.

**TABLE 3:** Main technical features of the five rem counters.

Rem counter	Manufacturer	Shape	Detector	Moderator	Energy range	Read‐out electronics
LINUS	In house	Spherical	Spherical ^3^He proportional counter	Polyethylene incorporating a boron‐doped rubber absorber and a 1cm thick lead shell	0.025 eV (thermal) to about 1 GeV	PDT20A‐SHV‐5 V module
LUPIN	ELSE NUCLEAR	Cylindrical	Cylindrical BF_3_ proportional counter	Polyethylene with lead and cadmium inserts	0.025 eV (thermal) to about 1 GeV	Logarithmic amplifier
Passive LINUS	In house	Spherical	PADC with ^10^B converter	Polyethylene with lead shell and cadmium inserts	0.025 eV (thermal) to about 1 GeV	–
WENDI II	Thermo Fisher Scientific	Cylindrical	Cylindrical ^3^He proportional counter	Polyethylene with a layer of tungsten powder	0.025 eV (thermal) to 5 GeV	RadEye PX
BIOREM	Thermo Fisher Scientific	Cylindrical	Cylindrical BF_3_ proportional counter	Polyethylene and boron carbide	0.025 eV (thermal) to 10 MeV	FH 40 G

### 2.3 Normalization of the results and experimental uncertainties

For each measurement, H*(10) was determined by Equation (1):

(1) $$H^{*}\left(10\right)=\frac{C\;\times \;k\;\times \;f_{p}}{N}\;$$

C represents the detector counts, k is the calibration factor given in Table 2, and f_p_ is a factor accounting for the positioning of the instruments. The factor f_p_ is considered equal to 1 and therefore does not affect the value of the measurement. Still, it is introduced in the equation to consider the precision of the instrument positioning when evaluating the measurement uncertainty. N represents the parameter used for the normalization of the results. The measured neutron doses were normalized to: (i) the number of primary particles delivered to the phantom; and (ii) the target dose in Gy. Normalization (i) is more intuitive for a direct comparison of secondary neutron field intensities and for intercomparing with MC results. The dose‐based normalization (ii) is more practical for comparing neutron doses from different ions and treatment plans for the same target volume, being consequently a normalization of clinical use/interest. All results reported in the next section are given with both normalisations. The different RBE of the ions was not considered in this study; this point is addressed in Section 4.1.

The standard uncertainty associated with each factor was evaluated as follows:

1. The counts (C) measured by the instrument follow a Poisson distribution, for which the relative standard deviation is calculated as $\frac{\sigma_{C}}{C}=\frac{1}{\sqrt{C}}$.
2. The relative standard uncertainty associated with the calibration factor (k) of each instrument is evaluated during the instrument calibration and mainly depends on the source used for the calibration. The values are reported in Table 2.
3. The impact of the instrument positioning (f_p_) is not trivial to evaluate. The neutron detectors were placed on stands to align the center of their active volumes with the isocentre. After each irradiation, the stands were moved around the room along with the detectors to the next measurement position. In‐room lasers were available to mark the isocentre. For the four measurement positions, only the height of the center of each rem counter was set according to the in‐room lasers. By measuring the distance to the isocentre of each position in the empty room, the accuracy of the instrument positioning was estimated to be 2cm as a conservative upper limit. For each irradiation, a relative uncertainty on detector positioning of 2% was assumed.
4. The factor N refers to either the number of ions or the dose delivered by the accelerator, according to the normalisation chosen. Its uncertainty depends on the accuracy and reproducibility of the treatment plan. The accelerator allows precise control of the beam parameters, guaranteeing that the treatment plan is reproducible with a relative uncertainty of 0.7% on the number of particles, which is directly linked to the dose in the treatment planning.

The combined standard uncertainty of the considered contributions was calculated assuming that these factors are uncorrelated.^53^ It should also be mentioned that the anisotropy of the cylindrical detectors (WENDI II, BIOREM, and LUPIN) was not considered in the evaluation of the uncertainties. These devices were calibrated for lateral (radial) irradiation, and their response can change up to 10% if the irradiation occurs in the axial direction.^49^ The rem counters were always placed vertically on the stands, as can be seen in Figure 1: most of the contribution is due to secondary radiation generated in the phantom and impinging radially on the detector. The axial component is only responsible for a minor contribution mainly due to wall, floor, and ceiling scattering.

### 2.4 Monte Carlo simulations and rem counters’ response to charged hadrons

MC simulations were run using a simplified geometry of the room. They were performed using MCNP (v. 6.2)^54^ and the CERN version of FLUKA (v. 4‐4.0)^55^, ^56^, ^57^ to compare inter‐code variability. The treatment room was modeled as a cube with outer dimensions of 6 m × 6 m × 6 m with 30cm thick concrete walls. The beam dump shown in Figure 1 was neglected, as its impact on the simulation results is negligible. The 60cm × 30cm × 30cm water phantom was placed at the centre of the room (which was filled with air). The scoring positions where the rem counters were placed were represented by spheres with a radius of 10cm filled with vacuum, whose centres were at the nominal distance and angle from the isocentre as sketched in Figure 1. The radiation source was a 10cm × 10cm expanded and aligned ion beam originating at 1 m from the surface of the phantom. This configuration was used to simulate the four primary ions, namely protons, ^4^He, ^12^C, and ^16^O ions. For each irradiation, the energy distribution of the source particles was retrieved from the accelerator log files, which record the number and energy of the delivered ions. The default cross sections, physics, and transport parameters of MCNP 6.2 were used, unless explicitly stated. For each simulation, the upper limit for neutron and ion energy was set to the maximum energy of the primary beam. For the FLUKA code, the PRECISIOn defaults were used. At energies of several hundred MeV per nucleon, the simulations strongly rely on physics models to represent the interaction between the beam and materials. For the MCNP code, the BERTINI^58^ model was used to describe the nuclear interactions above 20 MeV, whereas FLUKA exploits the PEANUT model.^55^, ^56^, ^57^

Both codes scored the following quantities:

- Neutron spectrum in each position,
- Neutron H*(10) in each position. This quantity was calculated by folding the scored fluence with the fluence to ambient dose equivalent conversion factors tabulated in ICRP 74,^37^
- Proton (p), deuteron (d), triton (t), helium‐3 nuclei (^3^He), and alpha particle (α) spectra in Position 1.

These quantities are the most useful ones to understand the response of the instruments in the radiation fields. For this reason, it was considered appropriate to have two independent simulations, from which the inter‐code variability could be evaluated.

Additionally, the neutron H*(10) in the room was scored with FLUKA, because of its convenient graphical user interface that allows for immediate plotting of the dose maps.

The results of the simulations are normalized to unit primary particle. The dosimetric quantity of interest can be calculated by multiplying the simulation results by the number of delivered ions, which is known from the log files, to be compared with the measurements. In addition, the absorbed dose at the isocentre was calculated with the MC codes and used to obtain data per unit Gy, also to be compared with the measurements.

While analyzing the experimental data, some anomalies were noticed suggesting that in the positions downstream of the phantom, the response of the rem counters was influenced by the presence of charged hadrons: light fragments other than neutrons generated inside the phantom by the interactions of the ion beam with water.

A full assessment of the response functions of rem counters to charged hadrons is beyond the scope of this work. Nevertheless, to better understand our results, the response of the LUPIN to the mixed radiation field emerging from the water phantom in the forward direction was simulated with MCNP. The MC simulations for ^12^C and ^16^O ions were repeated inserting in Position 1 the complete MC model of the LUPIN, and scoring the overall neutron counts in the active volume of the detector. The following quantities were scored:

- The expected counts measured by the detector,
- The relative contribution to the overall signal by each of the secondary charged particles. To do so, a filter was applied to the particles entering the detector volume, which allowed to isolate their contribution from the other particles.

Since the simulation of hadrons with energy above several tens of MeV per nucleon deeply relies on high‐energy physics models, two sets of simulations were run using the BERTINI and the Cascade‐Exciton (CEM) models.

## 3 RESULTS

### 3.1 Room monitoring of ambient dose equivalent

**FIGURE 3:**
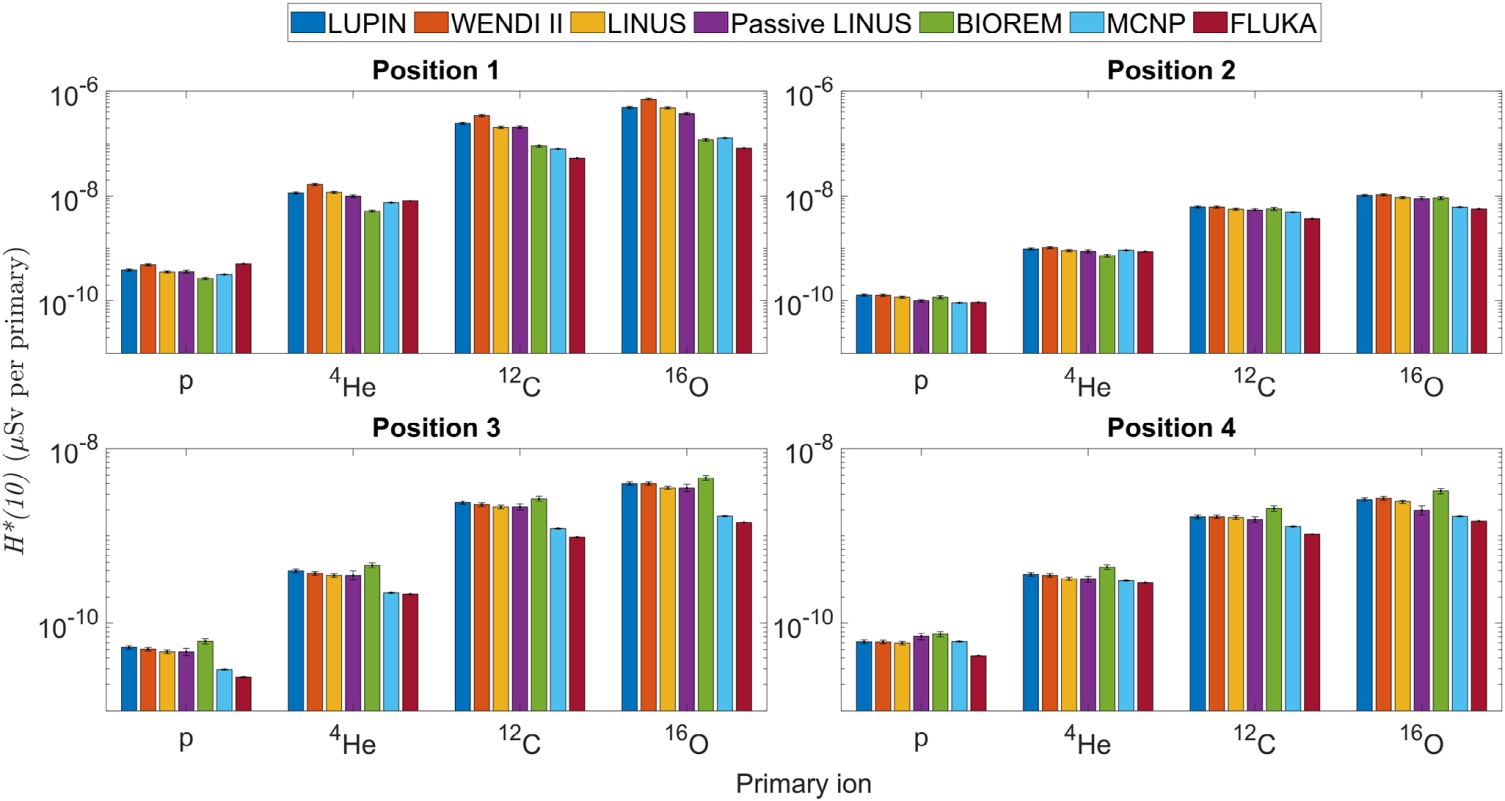
Ambient dose equivalent measured by the five rem counters and calculated via MC simulations in the four positions and for the four ions, normalised to primary particle. Note that the vertical scales for Positions 1–2 and Positions 3–4 are different.

**FIGURE 4:**
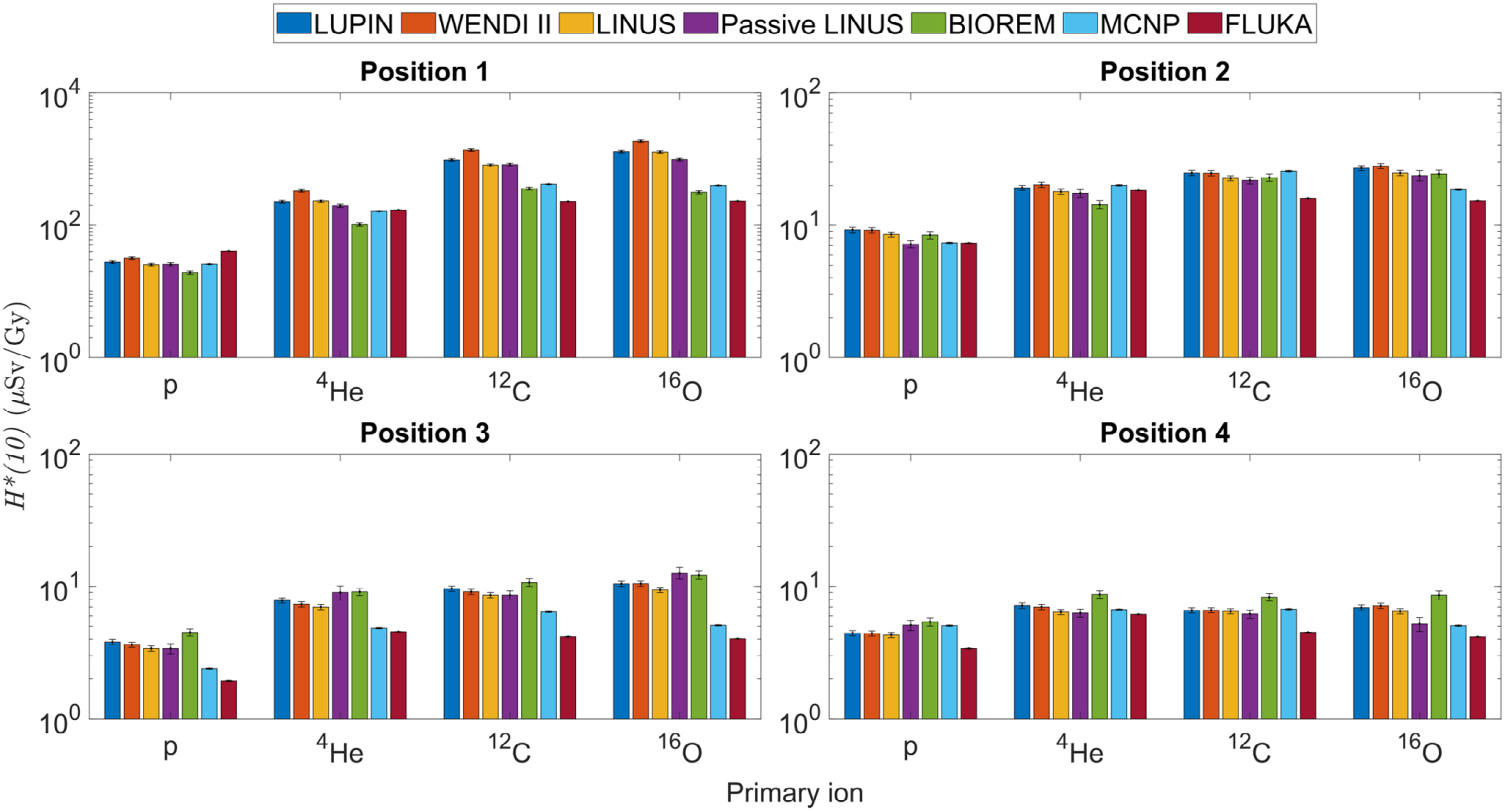
Ambient dose equivalent measured by the five rem counters and calculated via MC simulations in the four positions and for the four ions, normalised to target dose. Note that the vertical scale for Position 1 is different from the other three plots.

The experimental results are plotted in Figure 3 (normalization to primary particles) and in Figure 4 (normalization to target dose), along with the results of the MC simulations. The data for Position 1 are also listed in Tables 4 and 5, respectively, as these results present a particular interest and will be the subject of a more detailed analysis. The uncertainties correspond to one standard deviation (1σ). The numerical data for all positions can be found in the Supporting Information (Supplementary Material).

**TABLE 4:** Ambient dose equivalent measured by the five rem counters and calculated via MC simulations in Position 1 for the four ions, normalized to primary particle interacting within the water phantom.

H*(10) (µSv per primary) at Position 1							
Ion	LUPIN	WENDI II	LINUS	Pass. LINUS	BIOREM	MCNP	FLUKA
p	3.86 × 10^−10^ (1.8 × 10^−11^)	4.39 × 10^−10^ (1.4 × 10^−11^)	3.55 × 10^−10^ (1.6 × 10^−11^)	3.59 × 10^−10^ (2.6 × 10^−11^)	2.67 × 10^−10^ (9.7 × 10^−12^)	3.20 × 10^−10^ (3.2 × 10^−12^)	5.13 × 10^−10^ (1.0 × 10^−12^)
^4^He	1.14 × 10^−8^ (5.6 × 10^−10^)	1.67 × 10^−8^ (5.5 × 10^−10^)	1.17 × 10^−8^ (5.3 × 10^−10^)	1.00 × 10^−8^ (6.9 × 10^−10^)	5.18 × 10^−9^ (1.5 × 10^−10^)	7.46 × 10^−9^ (7.5 × 10^−11^)	8.09 × 10^−9^ (2.5 × 10^−12^)
^12^C	2.41 × 10^−7^ (1.1 × 10^−8^)	3.43 × 10^−7^ (1.1 × 10^−8^)	2.04 × 10^−7^ (9.2 × 10^−9^)	2.06 × 10^−7^ (1.4 × 10^−8^)	8.97 × 10^−8^ (2.5 × 10^−9^)	7.92 × 10^−8^ (7.9 × 10^−10^)	5.25 × 10^−8^ (2.6 × 10^−11^)
^16^O	4.93 × 10^−7^ (2.2 × 10^−8^)	7.11 × 10^−7^ (2.3 × 10^−8^)	4.86 × 10^−7^ (2.2 × 10^−8^)	3.72 × 10^−7^ (2.6 × 10^−8^)	1.19 × 10^−7^ (4.2 × 10^−9^)	1.28 × 10^−7^ (1.3 × 10^−9^)	8.17 × 10^−8^ (8.2 × 10^−11^)

The absolute uncertainties are given in brackets at one standard deviation.

The results show that the ambient dose equivalent due to secondary neutrons increases with the primary ion mass. This aspect is more significant for the forward Positions 1 and 2. Moreover, the trend of the results depends on the normalization used. Considering as an example the LINUS in Position 1, its reading increases by three orders of magnitude from hydrogen to oxygen if normalized to unit primary particle, whereas if normalized by the target dose the increase is only a factor of 40. The trend of the results normalized per primary particle is because the number of neutrons increases as the mass and the energy of the projectile increases. Instead, the results obtained by normalising to target dose are explained by the fact that the dose deposited by each primary particle in the target volume increases with the mass and atomic number of the particle. In other words, considerably less oxygen ions than protons are required to impart the same dose to the target.

Table 6 compares the results obtained with the MCNP and FLUKA codes, given in Figure 3. The MCNP simulations were stopped once a relative statistical uncertainty of 1% was reached, while the FLUKA simulations were run using 10^9^ primaries for protons and helium ions, and 10^8^ primaries for carbon and oxygen ions, allowing in all cases achieving relative uncertainties below 1%.

**FIGURE 5:**
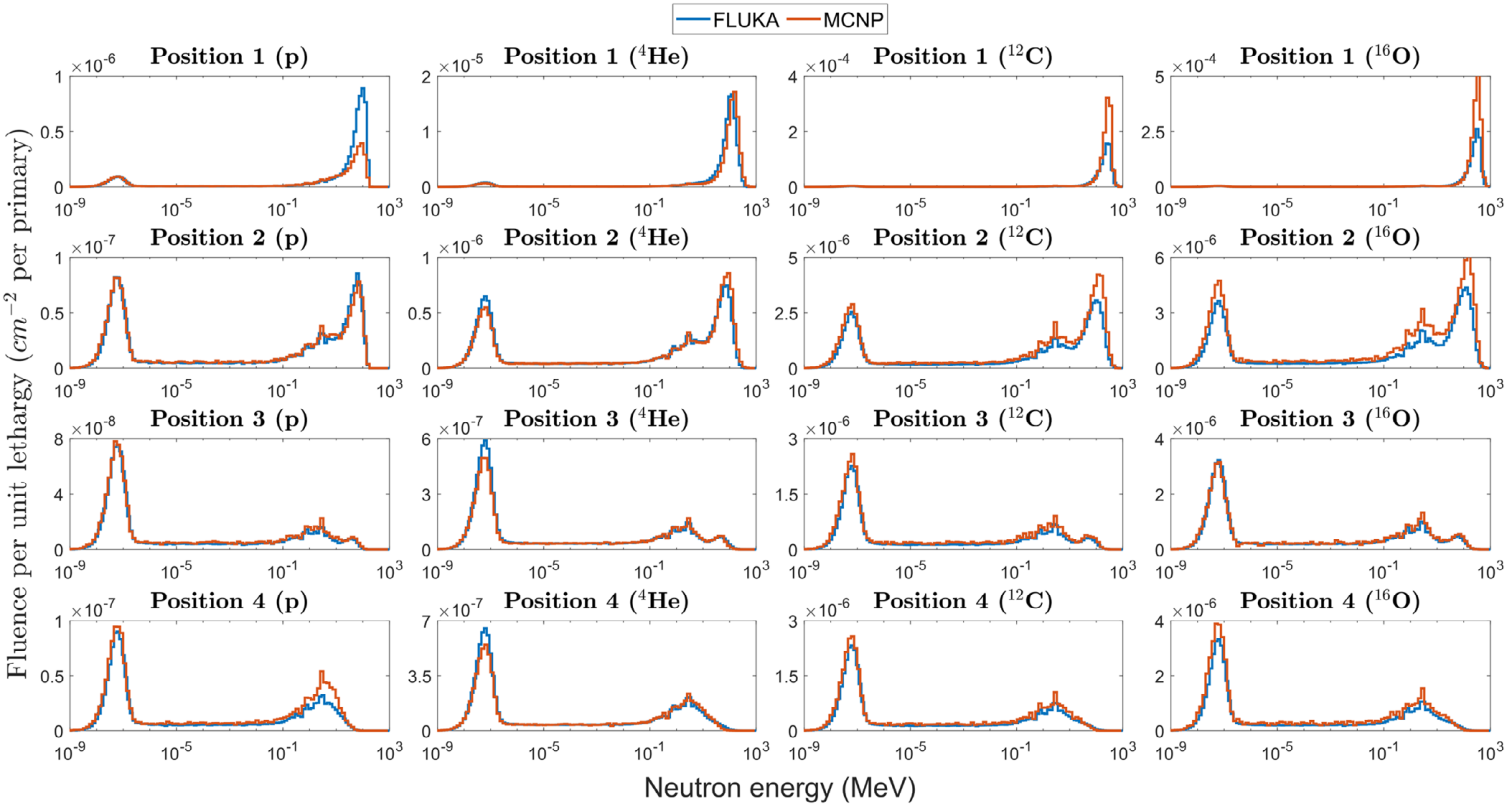
Neutron fluence spectra calculated with FLUKA and MCNP in the four experimental positions for the four ions.

Table 6 shows that the results from the two codes are in rather good agreement, with their ratio always within a factor of 1.6. It shall be noted that FLUKA systematically predicts a lower neutron H*(10) for carbon and oxygen ions. For the proton simulations, FLUKA predicts a lower neutron H*(10) in Positions 3 and 4, and a higher one in Position 1, whereas for Position 2 the codes agree well. An excellent agreement is found between the two codes in all positions for the simulations for helium ions, with discrepancies below 10%. To provide a better overview of the MC results, Figure 5 plots the neutron fluence spectra calculated by the two codes in the four measurement positions for the four ions.

The discrepancies between the two codes are generally more evident in Position 1, in which the high‐energy component of the field is dominant. This is mainly due to the differences in the physical models used by the codes to simulate the interactions of particles at these energies. For proton irradiation, FLUKA predicts a higher fluence of high‐energy neutrons in Position 1, and a lower one in Position 4, consistent with the results in Table 6. For helium irradiation, the main differences can be observed in the thermal region of the spectrum for Positions 2 to 4. However, low‐energy neutrons scarcely contribute to H*(10), and therefore, the estimated dosimetric quantity is approximately the same for both codes. Finally, for oxygen and carbon irradiations, MCNP always predicts higher neutron fluences. The results presented here are in line with the findings of previous studies.^13^, ^14^

**FIGURE 6:**
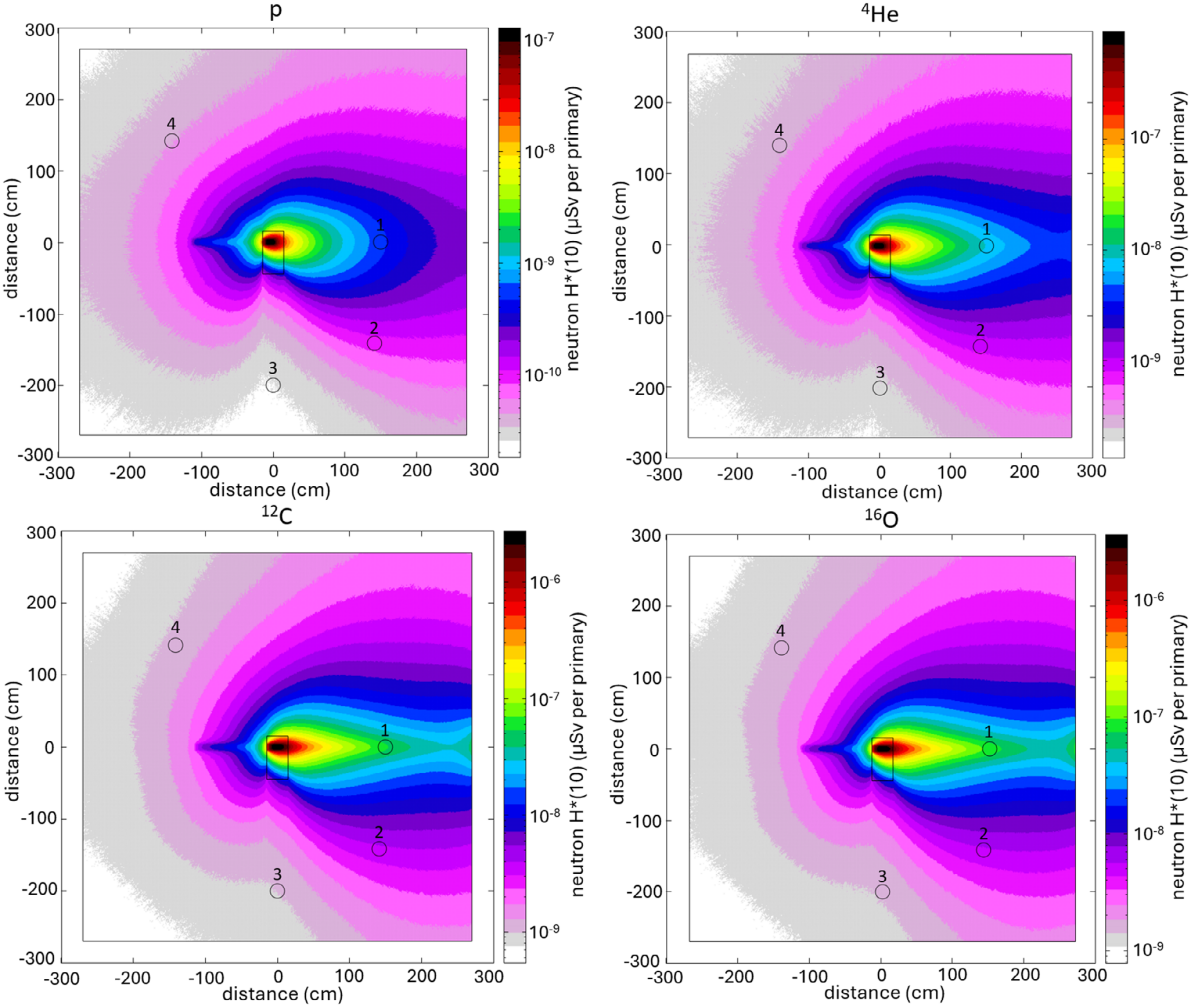
Neutron H*(10) map (in µSv per primary) in the experimental room for the four primary ions, calculated with the FLUKA code. The numbers refer to the rem counter positions shown in Figure 1.

Finally, Figure 6 shows the neutron H*(10) maps in the experimental room, in the horizontal plane passing through the centre of the phantom, calculated with FLUKA. As expected, the production of neutrons greatly increases from protons to heavier projectiles, with the largest dose in the forward direction. It should be noted that the H*(10) distribution is not symmetrical with respect to the beam direction because the beam does not impinge on the center of the phantom (Figure 1). The narrow “tail” of the distribution that is seen upstream of the phantom is associated with the primary ions’ path in air, whereas the shape of the dose distributions beyond the phantom is influenced by the neutron backscattering from the room back wall.

### 3.2 Rem counters’ response to charged hadrons

**FIGURE 7:**
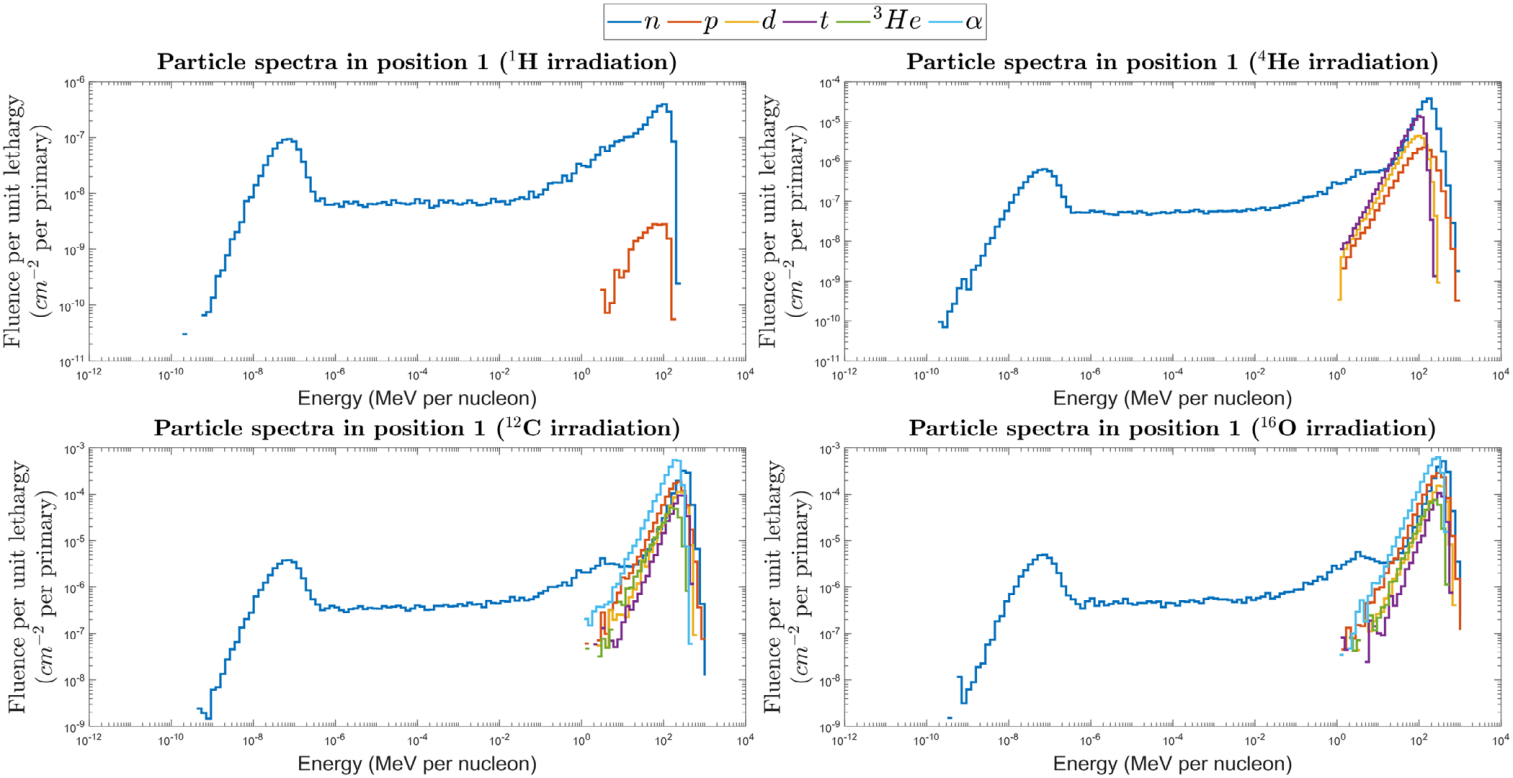
Particle spectra for the four ions in Position 1, given as fluence per primary, simulated using the MCNP code.

Figure 7 shows the particle spectra for the four primary ions in Position 1, limited to the secondary charged particles with a significant fluence, that is, fluence larger than 1% of the fluence of neutrons above 10 MeV. The spectra of secondary charged particles in Position 1 were calculated with both MCNP and FLUKA, obtaining similar spectra with a quantitative agreement between the two codes (in terms of integral fluence of secondary particles) always within a factor of 2. The differences in the spectra of secondary charged particles scored with the two codes are similar to those observed for the neutron spectra. This means that for proton irradiation, in which FLUKA scores a higher neutron fluence than MCNP, the fluences of secondary charged particles are also larger. Conversely, MCNP scores larger fluences of secondary charged particles for carbon and oxygen ions irradiations. As the following results will focus on simulations performed with MCNP only, Figure 7 shows the spectra simulated with this code. Regarding the other positions, the main contribution from secondary charged fragments is always from protons, whose fluence is at least one order of magnitude lower than that of neutrons. Hence, it is reasonable to assume that the contribution of secondary charged particles is only relevant in Position 1.

**FIGURE 8:**
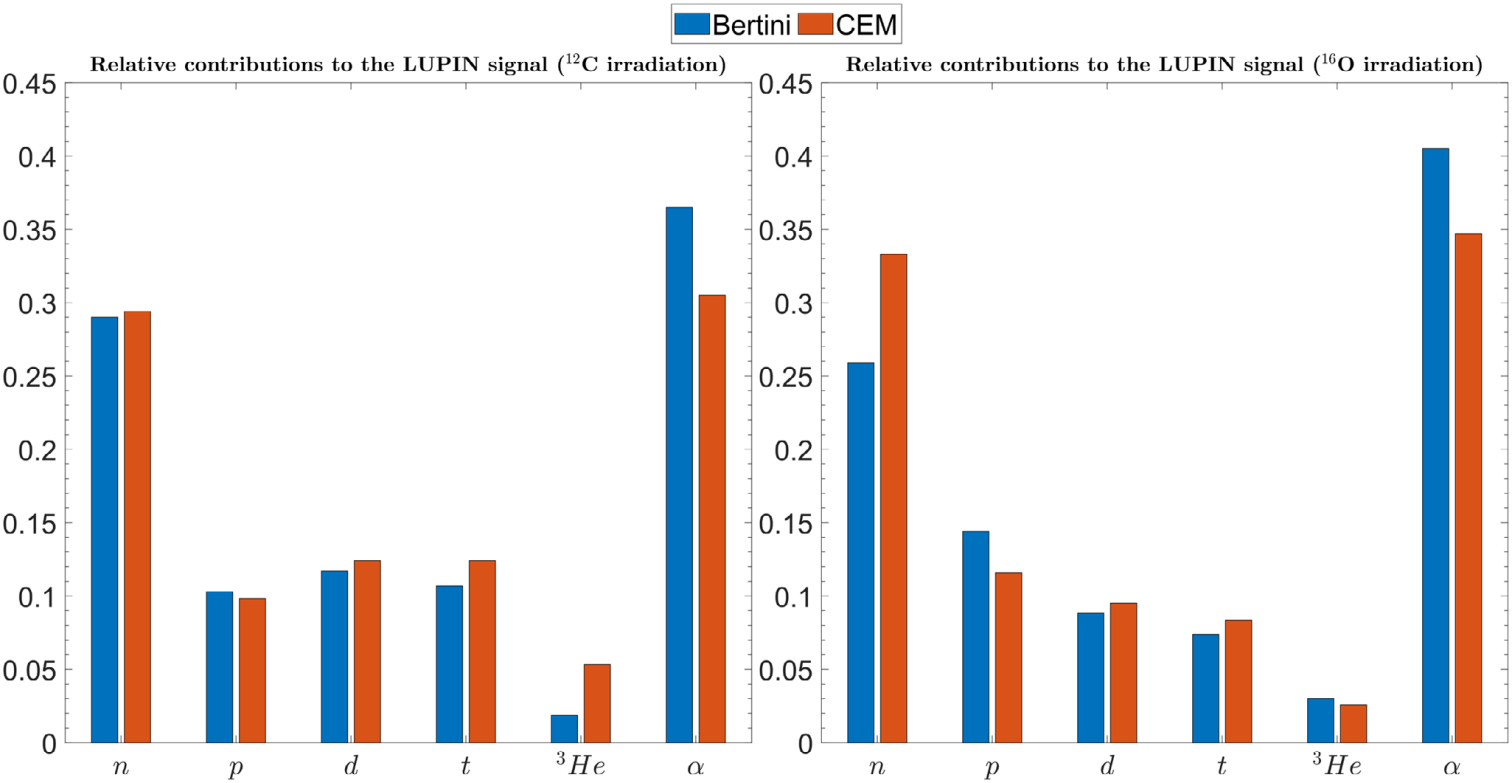
Relative contribution to the LUPIN reading of neutrons and charged fragments present in the forward direction in the mixed radiation field emerging from the water phantom, for irradiation with ^12^C (left) and ^16^O ions (right).

Moreover, for primary ^12^C and ^16^O, the sum of the secondary charged particles fluence is larger than the neutron fluence in Position 1. Hence, their contribution to the instrument response cannot be neglected. The response of the LUPIN in Position 1 to the mixed radiation field emerging from the water phantom is given in Table 7 as counts normalized to unit Gy. The relative contributions of the fragments to the overall response are reported in Figure 8. The results show that the rem counter counts are due to several types of hadrons, mostly α particles, in addition to neutrons. For ^12^C, both models estimate that approximately only 30% of the LUPIN counts are due to secondary neutrons. Instead, for ^16^O, the high‐energy physics model influences the results more evidently: BERTINI predicts that neutrons contribute 26% to the overall signal, whereas this percentage increases to 33% for CEM.

## 4 DISCUSSION

### 4.1 Room monitoring of ambient dose equivalent

All rem counters used in this experiment except the BIOREM have a response function that extends to the GeV region and therefore measure correctly in the HIT neutron field. All rem counters were calibrated with AmBe source neutrons: the calibration point is used to properly adjust the calibration curve that for each instrument had been determined by MC simulations and/or experimental measurements in appropriate calibration fields.

There is generally good agreement amongst all instruments, within about 15% for protons, 40% for ^4^He, ^12^C, and ^16^O ions in Position 1, within about 20% for protons in Position 2, and around 10% to 15% in all other cases. Nevertheless, some systematic discrepancies are observed in Tables 4 and 5, and Figures 3 and 4, for the WENDI II and the BIOREM. For the BIOREM, the results seem to indicate an overresponse of the instrument, as the measurements at Positions 3 and 4 overestimate the H*(10) with respect to the extended‐range rem counters (e.g., by about 30% as compared to the LINUS). In addition, it is well‐known that a conventional rem counter underestimates H*(10) by nearly 50% in a proton‐induced neutron field with an important high‐energy component as in Position 1 [see, e.g., past measurements at the CERN‐EU reference field (CERF) facility^39^, ^59^, ^60^], whereas here the BIOREM only slightly underestimates with respect to all other instruments for protons in Position 1. On the other hand, for all other ions in the same position the BIOREM severely underestimates as expected. This can be explained by the fact the fraction of the neutron spectrum above 10MeV increases with increasing ion mass. In addition, the response may be influenced by the presence of charged hadrons in the forward direction, as discussed below.

**TABLE 5:** Ambient dose equivalent measured by the five rem counters and calculated via MC simulations in Position 1 for the four ions, normalized to unit target dose.

H*(10) (µSv/Gy) at Position 1							
Ion	LUPIN	WENDI II	LINUS	Pass. LINUS	BIOREM	MCNP	FLUKA
p	27.8 (1.3)	31.6 (1.0)	25.5 (1.6)	25.8 (1.9)	19.2 (1.1)	25 (0.3)	41 (0.1)
^4^He	224.4 (10.2)	329.0 (10.8)	230.1 (10.4)	197.2 (13.7)	102.0 (5.5)	162 (1.6)	170 (0.1)
^12^C	963.3 (43.6)	1367.5 (44.8)	812.1 (36.8)	823.9 (56.4)	358.0 (19.0)	443 (4.4)	226 (0.1)
^16^O	1295.6 (58.7)	1870.0 (61.3)	1279.2 (57.9)	979.2 (67.2)	313.0 (17.9)	403 (4.0)	229 (0.2)

The absolute uncertainties are given in brackets at one standard deviation.

**TABLE 6:** Ratios of neutron H*(10) per primary ion impinging on the water phantom at the four measurement positions calculated with FLUKA and MCNP.

	FLUKA/MCNP			
	p	^4^He	^12^C	^16^O
Position 1	1.60	1.08	0.66	0.64
Position 2	1.02	0.94	0.77	0.87
Position 3	0.83	0.97	0.79	0.85
Position 4	0.69	0.95	0.82	0.89

Considering the WENDI II, it is known from previous works^59^, ^60^ that its response slightly overestimates (by about 10% to 15%) in the high‐energy region as compared to other extended‐range rem counters. This overestimation was observed in the presented results in Figures 3, 4, Tables 4 and 5. This effect is particularly evident with carbon and oxygen ions, since the energy of the secondary neutrons is expected to be higher.^14^ This may also be due to the contribution of the charged hadron component, as discussed below.

The presented results show that when normalized by the number of primary particles, the ambient dose equivalent increases with increasing primary ion mass and decreases with increasing angle to the primary beam (Figure 3). If normalized by the target dose, the H*(10) values in the two forward Positions 1 and 2 present a dependence on the primary ion whereas in Positions 3 and 4 the H*(10) are closer for all the ions (Figure 4). This is partly due to the change in the energy distribution of the secondary neutrons, with a relatively large high‐energy component compared to the thermal one in Positions 1 and 2, whereas in Positions 3 and 4, the thermal component is larger (Figure 5). Moreover, in Positions 1 and 2, the secondary charged fragments might also affect the response of the rem counters depending on the primary ion type.

The findings from this study contribute to the ongoing efforts of EURADOS to address the specific radiation protection needs of radiotherapy patients. The literature reports many results of measurements and calculations of out‐of‐field doses in hadron therapy but nearly all of them refer to proton facilities. Data are often not easy to compare as values of neutron H*(10) depend on distance from the neutron source, emission angle with respect to the beam direction, type of phantom, treatment plan, and variations in experimental conditions from one facility to another. Zorloni et al.^35^ analyzed the neutron H*(10) per unit therapy Gray at 90° from the beam axis and as a function of the distance from the isocentre from various studies, either measured or calculated, grouped according to beam delivery modality. At about 2 m from the isocentre, for active beam delivery systems, the H*(10) varies in the range from 0.3µSv/Gy to 5µSv/Gy. These values are consistent with those measured in the present experiment. With specific reference to previous EURADOS investigations, the measurements performed at the Trento Centro di Protonterapia (Trento, Italy)^26^ reported H*(10) values decreasing significantly with distance and angular position, in agreement with our results. We also confirmed their results that conventional rem counters underestimate H*(10) by up to a factor of 4, whereas the WENDI II was found to measure correctly (within 20%) over the entire energy range, again in agreement with our findings. The neutron H*(10) at a position approximately equivalent to Position 1 of the present experiment was about 50µSv/Gy, dropping to 8µSv/Gy, 3µSv/Gy, and 5µSv/Gy at positions approximately equivalent to our Positions 2, 3, and 4, respectively. The comparative measurements of scattered radiation in the Proteus C‐235 IBA facility (Cyclotron Centre Bronowice at the Institute of Nuclear Physics, CCB IFJ PAN, Kraków, Poland)^29^ showed, not surprisingly, that the lowest doses were measured at locations far from the phantom, and the highest doses at positions closest to it. The neutron H*(10) per unit target dose measured in the present experiment for protons are consistent with data collected by Van Hoey et al.^30^ at the Bronowice Cyclotron Center (CCB) Institute of Nuclear Physics (IFJ PAN) in Krakow (Poland) and the Skandion Clinic in Uppsala (Sweden), who reported between 3µSv/Gy and 300µSv/Gy at 1 m from the isocentre in the beam direction and between 0.5µSv/Gy and 50µSv/Gy at 1.5 m from the isocentre perpendicular to the beam direction. In the same investigations,^30^ an agreement within 30% between values of neutron H*(10) measured and simulated with MCNP, for the positions close to the phantom (where room‐scattered neutrons do not contribute significantly) was reported, close to what found in our experiment. In addition, a general decrease of neutron H*(10) with increasing distance from the target and higher neutron H*(10) in the forward direction as compared with the backward direction were observed. Mares et al.^33^ describe another experiment performed at CCB with three paediatric anthropomorphic phantoms (one, five, and ten years old) instead of a water phantom, irradiated with a simulated brain tumour treatment to evaluate whether parents could remain near their children during treatment. They also found that H*(10) values decreased as a function of distance and angular deviation with respect to the beam axis, with values varying in the range 0.1µSv/Gy 10µSv/Gy as a function of phantom employed, gantry angle and distance from the phantom that ranged from 1 m  to 2.25 m. The results of the rem counters intercomparison performed by EURADOS WG9 and WG11 in 2022^34^ are less directly comparable with our experiment because their measurements were performed at a Mevion S250 facility, where the cyclotron is mounted on the gantry and therefore generates more secondary neutrons in the treatment room compared to all other clinical facilities. Neutron H*(10) varied between 100µSv/Gy and 200µSv/Gy according to measurement position.

Only a few neutron H*(10) room‐mapping experiments are reported in the literature for carbon therapy. Yonai et al.^61^ performed measurements with a WENDI II at two carbon ion radiotherapy facilities with a 22cm × 24cm × 39cm water phantom, with the centre of the phantom coinciding with the isocentre. The measurement position was varied from 50 cm to 200cm, perpendicular to the ion beam direction. The study found that for passively scattered beams, the neutron H*(10) for 400 MeV/u carbon ions were similar to those for 160MeV passively scattered protons. At a location equivalent to Position 3 from our study (90° to the beam direction and 200cm from the isocentre), H*(10) ranged from 200µSv/Gy to 400µSv/Gy, depending on the irradiation field diameter.

In a subsequent study, Yonai et al.^62^ focused on neutron ambient dose equivalents in carbon ion radiotherapy using an active beam delivery system. Measurements were conducted using a similar setup as in their previous study^61^ to facilitate comparison. Their findings demonstrated that when assuming the same irradiation target, the neutron dose measured for the active beam delivery system was at most approximately 15% of that measured with a passive system, with this percentage decreasing as the distance from the isocentre increased. Moreover, it was shown that H*(10) for scanned carbon beams is similar to H*(10) for scanned proton beams. At a location equivalent to Position 3 from our study (90° to the beam direction and 200cm from the isocentre), measured neutron H*(10) values per primary particle ranged from 1.03 × 10^‐^^9^ µSv per primary to 1.26 × 10^‐^^9^ µSv per primary, depending on the scanning technique. In our study, for carbon ions at Position 3, the value measured with WENDI II was 2.29 × 10^‐^^9^ µSv per primary.

An additional point to be considered is the fact that the irradiation plans used in this study were designed to deliver a uniform physical dose in a cubic target inside a water phantom. This geometry is easier to implement into MC simulations, making the comparisons with experimental measurements more straightforward. When irradiation plans for patients are to be used, the RBE of the different primary ions comes into play, requiring using RBE‐optimized plans.^63^, ^64^, ^65^ The number of primary particles is thereby modified non‐linearly according to the primary ion RBE, the target geometry, and the desired target dose. For instance, carbon ions have a higher RBE than protons, resulting in even fewer primaries when comparing RBE‐optimized plans to physical dose‐optimized plans.^13^, ^14^, ^66^, ^67^, ^68^ Ultimately, since the number of primary particles changes, this influences the total number of secondary neutrons, affecting the trends presented.

The simplified MC simulations described here cannot exactly predict the dosimetric quantities at the various locations but are nonetheless useful to evaluate the order of magnitude of the neutron ambient dose equivalent. In general, the neutron dose at any given location in the room consists of a direct component emerging from the phantom and on a wall‐scattered one. The present simulations neglect the inner structure of the room and its precise dimensions; hence, they are inadequate to simulate the latter component correctly. Conversely, they should properly estimate the direct emission from the phantom. Therefore, the results may be realistic only for those positions in which the direct component dominates. Since the neutron emission from the phantom strongly depends on the angle with the primary beam and is mainly forward directed, it is reasonable to expect that the most realistic values are for Position 1.

The trends observed with the MC results follow the trends of the rem counters measurements. Nevertheless, both MCNP and FLUKA systematically underestimate the H*(10) measured by the rem counters in Positions 3 and 4. This is because in these positions an accurate representation of the room would be required to account for the low‐energy neutron component in the simulations. Instead, in Positions 1 and 2 the cascade neutrons emitted from the target dominate, but the predictions from the MC codes are still (well) below the measured values, in particular for ^12^C and ^16^O ions: about 30 4% in Position 2 and a factor of 3 to 4 in Position 1. Therefore, a dedicated analysis of the production of secondary fragments, neutrons and charged particles, and their influence on the rem counter response, was carried out to understand this inconsistency.

### 4.2 Rem counters’ response to charged hadrons

The results in Tables 4 and 5 and Figures 3 and 4 show a rather good agreement, for radiation protection purposes, between simulation results and experimental data in Position 1 for protons and ^4^He ions: depending on the rem counter and MC code, within 40% for protons and about a factor of 2 for ^4^He ions. In this position, for ^12^C and ^16^O ions the MC simulations underestimate the measured values. This probably depends on the light fragments mixed with neutrons that are present downstream of the phantom. For protons and helium ions, the secondary radiation field is dominated by neutrons. For carbon and oxygen ions, it has been shown above that there is a relatively large number of light fragments in the secondary field, especially α particles. It is reasonable to assume that rem counters are sensitive to light fragments, as shown in past measurements,^69^ but the response of moderator‐type instruments to charged hadrons has not been generally investigated in the literature.

In our results, the MC predictions of the measured counts are fairly accurate, as reported in Table 7. Conversely, as shown in Tables 4 and 5, in Position 1, the neutron H*(10) predicted by MCNP is lower, by a factor of 3 to 4, than the experimental data for both carbon and oxygen ions. This suggests that the rem counter readings are substantially affected by the charged particles present in the secondary radiation field and interacting with the moderator or depositing energy directly into the active volume, leading to an overestimation of the neutron ambient dose equivalent.

**TABLE 7:** Comparison between the measured counts of the LUPIN (normalized to unit Gy at the isocentre) and the MCNP predictions for the ^12^C and ^16^O beam irradiations.

Ion	Measured	MCNP—CEM	Deviation	MCNP—BERTINI	Deviation
^12^C	1.61 × 10^6^	1.82 × 10^6^	13%	1.88 × 10^6^	17%
^16^O	2.16 × 10^6^	2.23 × 10^6^	4%	1.99 × 10^6^	−8%

The deviations given in the fourth and sixth columns are the percent difference (MCNP‐measured)/measured.

Based on the results presented in Figure 8, it is possible to estimate the “real” in‐room neutron H*(10) measured by the LUPIN for primary ^12^C and ^16^O ions by computing a corrected number of counts accounting only for the relative contribution of the secondary neutrons to the instrument reading. To illustrate this, Table 8 compares the simulated neutron H*(10) and the LUPIN measurements from Table 5, with the instrument reading corrected for the influence of the charged particles on the signal. This shows the feasibility of introducing MC‐based corrections to account for the charged hadrons in the response of the rem counters.

**TABLE 8:** Comparison between the simulated neutron H*(10) and the LUPIN measurements (from Table 4), with its reading corrected for the charged particles’ influence on the signal.

Ion	MCNP	LUPIN (corrected—BERTINI)	Deviation	LUPIN (corrected—CEM)	Deviation
		(µSv per primary)		(µSv per primary)	
^12^C	7.92 × 10^−8^	6.99 × 10^−8^	13%	7.11 × 10^−8^	11%
^16^O	1.28 × 10^−7^	1.28 × 10^−7^	0%	1.63 × 10^−7^	−21%

The columns “LUPIN (corrected—BERTINI)” and “LUPIN (corrected—CEM)” refer to the different high‐energy physics models used for the correction. The deviations given in the fourth and sixth columns are the percent difference (MCNP‐measured)/measured.

A full characterization of the charged hadron component mixed with the neutron field downstream of the patient (or phantom), generated by ions heavier than ^4^He, will require a dedicated experiment and instrumentation capable of discriminating the various particles. Instrumentation or dosimeters suitable for these measurements may be, for example, Tissue Equivalent Proportional Counters (TEPC),^70^ silicon detectors,^36^ the Medipix hybrid pixel detector,^71^ or gas detectors like the GEMPix.^72^, ^73^

## 5 CONCLUSION

This article has reported on in‐room neutron H*(10) measurements at the experimental room of the Heidelberg Ion Beam Therapy Center (HIT), for the same virtual target in a water phantom irradiated with the four primary ions currently available. The measurements were performed with five rem counters of different design, four extended‐range instruments and one conventional unit with response limited to a few MeV. The two results of this experiment are: (1) the characterization of the neutron field at various locations in the room, for the four ions; and (2) the evidence of a potential problem with the use of moderator‐type instrumentation in a mixed radiation field where charged hadrons are present in addition to neutrons.

The results of the measurements with the four extended‐range instruments agree well, whereas the conventional rem counter underestimates H*(10) in a neutron field with a large high‐energy component. The experimental results, complemented by MC simulations with FLUKA and MCNP, have shown that the intensity of the neutron field increases with primary ion mass and that this trend is more significant in the forward direction. The amount of this increase is a function of the normalisation employed (number of primary particles or dose delivered to the target) because of the interplay between neutron production by a given ion at a given energy and dose deposited by each ion in the target. Looking toward the clinical implications, the trend of these results may be different if RBE‐optimized irradiation plans are used.

Overall, the two MC codes provide consistent predictions, within a factor of 1.6 for the downstream position and lower differences in the other cases, and agree with the experimental data with some exceptions. FLUKA predicts a lower neutron H*(10) than MCNP for ^12^C and ^16^O ions, whereas for protons it predicts a lower H*(10) in the lateral and backward positions, and a higher H*(10) in the forward direction. An excellent agreement is found between the two codes in all positions for ^4^He ions, with discrepancies below 10 %.

The experimental results combined with the MC simulations also showed that neutrons are not necessarily the only secondary radiation field to be monitored. This has been here confirmed and clarified by dedicated MC simulations designed to better understand the rem counters’ response in the presence of heavy charged fragments. This component mainly affects the radiation field downstream, opening the issue on whether the use of rem counters, and in general moderator‐type instruments, is still feasible in neutron fields with a relatively high presence of charged hadrons. It may still be the case, but it will require dedicated studies, both simulations and experiments, to characterize the response of an instrument to the non‐neutron components (as done in a past work for a Bonner Sphere Spectrometer), and to gain complete knowledge of the types and energies of the charged hadrons mixed with the neutron field downstream of the phantom. The European Radiation Dosimetry Group (EURADOS) is currently in the designing phase of such an experiment, which is tentatively planned for fall 2025.

## CONFLICT OF INTEREST STATEMENT

The authors declare no conflicts of interest.

## Supporting information




Supporting Information

## References

[1] Dosanjh M , Bernier J . Advances in Particle Therapy: A Multidisciplinary Approach. 2018.

[2] accessed on February 4 2025. https://www.ptcog.site/

[3] Kraan A , Del Guerra A . Technological developments and future perspectives in particle therapy: a topical review. IEEE Trans Radiat Plasma Med Sci. 2024:1. doi:10.1109/TRPMS.2024.3372189

[4] accessed on 4 February 2025. https://www.cnao.it

[5] accessed on February 4 2025. https://www.klinikum.uni‐heidelberg.de/interdisziplinaere‐zentren/heidelberger‐ionenstrahl‐therapiezentrum‐hit

[6] accessed on February 4 2025. https://www.medaustron.at/en/

[7] Haberer T , Becher W , Schardt D , Kraft G . Magnetic scanning system for heavy ion therapy. Nucl Instrum Methods Phys Res A. 1993;330(1):296‐305. doi:10.1016/0168-9002(93)91335-K

[8] Haberer T , Debus J , Eickhoff H , Jäkel O , Schulz‐Ertner D , Weber U . The Heidelberg ion therapy center. Radiother Oncol. 2004;73:S186‐S190. doi:10.1016/S0167-8140(04)80046-X 15971340

[9] Jäkel O , Kraft G , Karger CP . The history of ion beam therapy in Germany. Z Med Phys. 2022;32(1):6‐22. doi:10.1016/j.zemedi.2021.11.003 35101337 PMC9948864

[10] Mairani A , Mein S , Blakely E , et al. Roadmap: helium ion therapy. Phys Med Biol. 2022;67(15):15TR02. doi:10.1088/1361-6560/ac65d3 35395649

[11] Wickert R , Tessonnier T , Deng M , et al. Radiotherapy with helium ions has the potential to improve both endocrine and neurocognitive outcome in pediatric patients with ependymoma. Cancers (Basel). 2022;14(23):5865. doi:10.3390/cancers14235865 36497348 PMC9736041

[12] Tessonnier T , Ecker S , Besuglow J , et al. Commissioning of helium ion therapy and the first patient treatment with active beam delivery. Int J Radiat Oncol Biol Phys. 2023;116(4):935‐948. doi:10.1016/j.ijrobp.2023.01.015 36681200

[13] Vedelago J , Geser FA , Muñoz ID , Stabilini A , Yukihara EG , Jäkel O . Assessment of secondary neutrons in particle therapy by Monte Carlo simulations. Phys Med Biol. 2022;67(1):015008. doi:10.1088/1361-6560/ac431b 34905742

[14] Geser FA , Stabilini A , Christensen JB , et al. A Monte Carlo study on the secondary neutron generation by oxygen ion beams for radiotherapy and its comparison to lighter ions. Phys Med Biol. 2024;69(1):015027. doi:10.1088/1361-6560/ad0f45 37995363

[15] Glowa C , Saager M , Hintz L , et al. Relative biological effectiveness of oxygen ion beams in the rat spinal cord: dependence on linear energy transfer and dose and comparison with model predictions. Phys Imaging Radiat Oncol. 2024;30:100581. doi:10.1016/j.phro.2024.100581 38711920 PMC11070926

[16] Hall EJ . Intensity‐modulated radiation therapy, protons, and the risk of second cancers. Int JRadiat Oncol Biol Phys. 2006;65(1):1‐7. doi:10.1016/j.ijrobp.2006.01.027 16618572

[17] Zacharatou Jarlskog C, Paganetti H . Risk of developing second cancer from neutron dose in proton therapy as function of field characteristics, organ, and patient age. Int JRadiat Oncol Biol Phys. 2008;72(1):228‐235. doi: 10.1016/j.ijrobp.2008.04.069 18571337

[18] Schneider U , Hälg R . The impact of neutrons in clinical proton therapy. Front Oncol. 2015;5:235. doi:10.3389/fonc.2015.00235 26557501 PMC4617104

[19] Hälg RA , Schneider U . Neutron dose and its measurement in proton therapy—current state of knowledge. Br J Radiol. 2019:20190412. doi:10.1259/bjr.20190412 PMC7066952 31868525

[20] Newhauser WD , Durante M . Assessing the risk of second malignancies after modern radiotherapy. Nat Rev Cancer. 2011;11(6):438‐448. doi:10.1038/nrc3069 21593785 PMC4101897

[21] Gottschalk B . Neutron dose in scattered and scanned proton beams: in regard to Eric J. Hall (Int J Radiat Oncol Biol Phys 2006;65:1‐7). 2006;66(5):1594. doi:10.1016/j.ijrobp.2006.08.014 17126218

[22] Paganetti H , Bortfeld T , Delaney TF . Neutron dose in proton radiation therapy: in regard to Eric J. Hall (Int J Radiat Oncol Biol Phys 2006;65:1‐7). Int J Radiat Oncol Biol Phys. 2006;66(5):1594‐1595. doi:10.1016/j.ijrobp.2006.08.005 17126219

[23] Carnicer A , Letellier V , Rucka G , Angellier G , Sauerwein W , Herault J . Study of the secondary neutral radiation in proton therapy: toward an indirect in vivo dosimetry. Phys Med. 2013;29:e18. doi:10.1016/j.ejmp.2013.08.058 23231280

[24] Vedelago J , Schmidt S , Stengl C , Karger CP , Jäkel O . Secondary neutrons in proton and light ion beam therapy: a review of current status, needs and potential solutions. Radiat Meas. 2024;176:107214. doi:10.1016/j.radmeas.2024.107214

[25] accessed on February 4 2025. https://eurados.sckcen.be/

[26] Farah J , Mares V , Romero‐Expósito M , et al. Measurement of stray radiation within a scanning proton therapy facility: eURADOS WG9 intercomparison exercise of active dosimetry systems. Med Phys. 2015;42(5):2572‐2584. doi:10.1118/1.4916667 25979049

[27] Knežević Ž , Ambrozova I , Domingo C , et al. Comparison of response of passive dosimetry systems in scanning proton radiotherapy—a study using pediatric anthropomorphic phantoms. Radiat Prot Dosimetry. 2018;180(1‐4):256‐260. doi:10.1093/rpd/ncx254 29165619

[28] Stolarczyk L , Trinkl S , Romero‐Expósito M , et al. Dose distribution of secondary radiation in a water phantom for a proton pencil beam—EURADOS WG9 intercomparison exercise. Phys Med Biol. 2018;63(8):085017. doi:10.1088/1361-6560/aab469 29509148

[29] Wochnik A , Stolarczyk L , Ambrožová I , et al. Out‐of‐field doses for scanning proton radiotherapy of shallowly located paediatric tumours—a comparison of range shifter and 3D printed compensator. Phys Med Biol. 2021;66(3):035012. doi:10.1088/1361-6560/abcb1f 33202399

[30] Van Hoey O , Stolarczyk L , Lillhök J , et al. Simulation and experimental verification of ambient neutron doses in a pencil beam scanning proton therapy room as a function of treatment plan parameters. Front Oncol. 2022;12:903537. doi:10.3389/fonc.2022.903537 36158693 PMC9494550

[31] Knežević Ž , Stolarczyk L , Ambrožová I , et al. Out‐of‐field doses produced by a proton scanning beam inside pediatric anthropomorphic phantoms and their comparison with different photon modalities. Front Oncol. 2022;12:904563. doi:10.3389/fonc.2022.904563 35957900 PMC9361051

[32] Majer M , Ambrožová I , Davídková M , et al. Out‐of‐field doses in pediatric craniospinal irradiations with 3D‐CRT, VMAT, and scanning proton radiotherapy: a phantom study. Med Phys. 2022;49(4):2672‐2683. doi:10.1002/mp.15493 35090187

[33] Mares V , Farah J , De Saint‐Hubert M , et al. Neutron radiation dose measurements in a scanning proton therapy room: can parents remain near their children during treatment? Front Oncol. 2022;12:903706. doi:10.3389/fonc.2022.903706 35912238 PMC9330633

[34] Zorloni G , Bosmans G , Brall T , et al. Joint EURADOS WG9‐WG11 rem‐counter intercomparison in a Mevion S250i proton therapy facility with Hyperscan pulsed synchrocyclotron. Phys Med Biol. 2022;67(7):075005. doi:10.1088/1361-6560/ac5b9c 35259730

[35] Zorloni G , Bosmans G , Brall T , et al. EURADOs Rem‐Counter intercomparison at Maastro proton therapy centre: comparison with literature data. Radiat Prot Dosimetry. 2022;198(19):1471‐1475. doi:10.1093/rpd/ncac189 36138419

[36] Knoll GF . Radiation detection and measurement. Proc IEEE. 1981;69:495‐495. doi:10.1109/PROC.1981.12016

[37] ICRP Publication 74: Conversion Coefficients for Use in Radiological Protection against External Radiation. SAGE Publications; 1997. doi:10.1016/S0146-6453(96)90010-X

[38] Birattari C , Ferrari A , Nuccetelli C , Pelliccioni M , Silari M . An extended range neutron rem counter. Nucl Instrum Methods Phys Res A. 1990;297(1):250‐257. doi:10.1016/0168-9002(90)91373-J

[39] Birattari C , Esposito A , Ferrari A , Pelliccioni M , Silari M . A neutron survey meter with sensitivity extended up to 400 MeV. Radiat Prot Dosimetry. 1992;44:193‐197. doi:10.1093/rpd/44.1-4.193

[40] Birattari C , Esposito A , Ferrari A , Pelliccioni M , Silari M . Calibration of the neutron rem counter LINUS in the energy range from thermal to 19 MeV. Nucl Instrum Methods Phys Res A. 1993;324(1):232‐238. doi:10.1016/0168-9002(93)90982-N

[41] Birattari C , Esposito A , Ferrari A , Pelliccioni M , Rancati T , Silari M . The extended range neutron Rem counter LINUS: overview and latest developments. Radiat Prot Dosimetry. 1998;76(3):135‐148. doi:10.1093/oxfordjournals.rpd.a032258

[42] Accessed February 4, 2025. https://pdt‐inc.com/products/10series/10series_datasheet/index.html

[43] Ferrarini M , Varoli V , Favalli A , Caresana M , Pedersen B . A wide dynamic range BF3 neutron monitor with front‐end electronics based on a logarithmic amplifier. Nucl Instrum Methods Phys Res A. 2010;613(2):272‐276. doi:10.1016/j.nima.2009.11.078

[44] Caresana M , Ferrarini M , Manessi GP , Silari M , Varoli V . LUPIN, a new instrument for pulsed neutron fields. Nucl Instrum Methods Phys Res A. 2013;712:15‐26. doi:10.1016/j.nima.2013.01.060

[45] Agosteo S , Caresana M , Ferrarini M , Silari M . A passive rem counter based on CR39 SSNTD coupled with a boron converter. Radiat Meas. 2009;44(9):985‐987. doi:10.1016/j.radmeas.2009.10.053

[46] Agosteo S , Caresana M , Ferrarini M , Silari M . A dual‐detector extended range rem‐counter. Radiat Meas. 2010;45:1217‐1219. doi:10.1016/j.radmeas.2010.05.002

[47] Caresana M , Ferrarini M , Parravicini A , Naik SA. Calibration of a passive rem counter with monoenergetic neutrons. Radiat Meas. 2014;71:498‐501. doi:10.1016/j.radmeas.2014.07.019

[48] Mi.am Srl, Politrack—User Manual, CR‐39 Fast neutrons—CR‐39 Thermal neutrons, v. 6.9, (2023).

[49] Olsher R , Hsu H , Beverding A , et al. WENDI: an improved neutron rem meter. Health Phys. 2000;79:170‐181. doi:10.1097/00004032-200008000-00010 10910387

[50] Martínez‐Francés E , et al. Neutron detectors in proton therapy: calibration, operational in situ verification and comparison with Monte Carlo simulations. Radiat PhysChem. 2025;228:112362. doi:10.1016/j.radphyschem.2024.112362

[51] Perl J , Shin J , Schümann J , Faddegon B , Paganetti H . TOPAS: an innovative proton Monte Carlo platform for research and clinical applications. Med Phys. 2012;39(11):6818‐6837. doi:10.1118/1.4758060 23127075 PMC3493036

[52] Caresana M , Denker A , Esposito A , et al. Intercomparison of radiation protection instrumentation in a pulsed neutron field. Nucl Instrum Methods Phys Res A. 2014;737:203‐213. doi:10.1016/j.nima.2013.11.073

[53] Metrology JointCommitteeforGuidesin . 2008. https://www.bipm.org/documents/20126/2071204/JCGM_100_2008_E.pdf. Evaluation of Measurement Data — Guide to the Expression of Uncertainty in Measurement.

[54] Werner CJ , Armstrong JC , Brown FB , et al. 2017. http://permalink.lanl.gov/object/tr?what=info:lanl‐repo/lareport/LA‐UR‐17‐29981

[55] accessed on February 4 2025. https://fluka.cern

[56] Ahdida C , Bozzato D , Calzolari D , et al. New capabilities of the FLUKA multi‐purpose code. Front Phys. 2022;9. doi:10.3389/fphy.2021.788253

[57] Battistoni G , Boehlen T , Cerutti F , et al. Overview of the FLUKA code. Ann Nucl Energy. 2015;82:10‐18. doi:10.1016/j.anucene.2014.11.007

[58] Bertini HW . Low‐energy intranuclear cascade calculation. Phys Rev. 1963;131(4):1801‐1821. doi:10.1103/PhysRev.131.1801

[59] Birattari C , Esposito A , Fasso A , et al. Intercomparison of the response of dosemeters used in high energy stray radiation fields. Radiat Prot Dosimetry. 1994;51(2):87‐94. doi:10.1093/oxfordjournals.rpd.a082125

[60] Caresana M , Helmecke M , Kubancak J , et al. Instrument intercomparison in the high‐energy mixed field at the CERN‐EU reference field (CERF) facility. Radiat Prot Dosimetry. 2013;161:67‐72. doi:10.1093/rpd/nct312 24292486

[61] Yonai S , Matsufuji N , Kanai T , et al. Measurement of neutron ambient dose equivalent in passive carbon‐ion and proton radiotherapies. Med Phys. 2008;35:4782‐4792. doi:10.1118/1.2989019 19070210

[62] Yonai S , Furukawa T , Inaniwa T . Measurement of neutron ambient dose equivalent in carbon‐ion radiotherapy with an active scanned delivery system. Radiat Prot Dosimetry. 2014;161(1‐4):433‐436. doi:10.1093/rpd/nct251 24126486

[63] Schardt D , Elsässer T , Schulz‐Ertner D . Heavy‐ion tumor therapy: physical and radiobiological benefits. Rev Mod Phys. 2010;82:383‐425. doi:10.1103/RevModPhys.82.383

[64] Bronk L , Guan F , Patel D , et al. Mapping the relative biological effectiveness of proton, helium and carbon ions with high‐throughput techniques. Cancers (Basel). 2020;12(12):3658. doi:10.3390/cancers12123658 33291477 PMC7762185

[65] Jäkel O . Physical advantages of particles: protons and light ions. Br J Radiol. 2020;93(1107):20190428. doi:10.1259/bjr.20190428 31556333 PMC7066975

[66] Wilkens JJ , Oelfke U . Direct comparison of biologically optimized spread‐out Bragg peaks for protons and carbon ions. Int J Radiat Oncol Biol Phys. 2008;70(1):262‐266. doi:10.1016/j.ijrobp.2007.08.029 17935903

[67] Elsässer T , Weyrather WK , Friedrich T , et al. Quantification of the relative biological effectiveness for ion beam radiotherapy: direct experimental comparison of proton and carbon ion beams and a novel approach for treatment planning. Int J Radiat Oncol Biol Phys. 2010;78(4):1177‐1183. doi:10.1016/j.ijrobp.2010.05.014 20732758

[68] Karger CP , Glowa C , Peschke P , Kraft‐Weyrather W . The RBE in ion beam radiotherapy: in vivo studies and clinical application. Z Med Phys. 2021;31(2):105‐121. doi:10.1016/j.zemedi.2020.12.001 33568337

[69] Agosteo S , Dimovasili E , Fasso A , Silari M . The response of a bonner sphere spectrometer to charged hadrons. Radiat Prot Dosimetry. 2004;110:161‐168. doi:10.1093/rpd/nch187 15353640

[70] Agosteo S . Detectors for measurement of microdosimetric quantities. Radiat Meas. 2022;156:106807. doi:10.1016/j.radmeas.2022.106807

[71] Medipix Collaboration . accessed on February 4 2025. https://medipix.web.cern.ch/

[72] Leidner J , Ciocca M , Mairani A , Murtas F , Silari M . A GEMPix‐based integrated system for measurements of 3D dose distributions in water for carbon ion scanning beam radiotherapy. Med Phys. 2020;47(6):2516‐2525. doi:10.1002/mp.14119 32135033 PMC7384041

[73] Leidner J , Murtas F , Silari M . Medical applications of the GEMPix. Appl Sci. 2021;11(1):440. doi:10.3390/app11010440

