# Supplementary material for: Out‐of‐field neutron radiation from clinical proton, helium, carbon, and oxygen ion beams
Source: Med Phys. 2025 Apr 4;52(6):4924–40. doi: 10.1002/mp.17797 (PMC12149714; doi:10.1002/mp.17797)
Supplement: Supplementary file 1 — Supporting Information [file MP-52-4924-s001.pdf]

SUPPLEMENTARY MATERIAL

Table S1: Ambient dose equivalent measured by the five rem counters and calculated via MC simulations in the four positions and for the four ions, normalised to primary particle interacting within the water phantom. The absolute uncertainties are given in brackets at one standard deviation.

**$H^*(10)$ (μSv per primary) at Position 1**							
**Ion**	**LUPIN**	**WENDI II**	**LINUS**	**Pass. LINUS**	**BIOREM**	**MCNP**	**FLUKA**
**p**	3.86×10^{-10} (1.8×10^{-11})	4.39×10^{-10} (1.4×10^{-11})	3.55×10^{-10} (1.6×10^{-11})	3.59×10^{-10} (2.6×10^{-11})	2.67×10^{-10} (9.7×10^{-12})	3.20×10^{-10} (3.2×10^{-12})	5.13×10^{-10} (1.0×10^{-12})
**^{4}He**	1.14×10^{-8} (5.6×10^{-10})	1.67×10^{-8} (5.5×10^{-10})	1.17×10^{-8} (5.3×10^{-10})	1.00×10^{-8} (6.9×10^{-10})	5.18×10^{-9} (1.5×10^{-10})	7.46×10^{-9} (7.5×10^{-11})	8.09×10^{-9} (2.5×10^{-12})
**^{12}C**	2.41×10^{-7} (1.1×10^{-8})	3.43×10^{-7} (1.1×10^{-8})	2.04×10^{-7} (9.2×10^{-9})	2.06×10^{-7} (1.4×10^{-8})	8.97×10^{-8} (2.5×10^{-9})	7.92×10^{-8} (7.9×10^{-10})	5.25×10^{-8} (2.6×10^{-11})
**^{16}O**	4.93×10^{-7} (2.2×10^{-8})	7.11×10^{-7} (2.3×10^{-8})	4.86×10^{-7} (2.2×10^{-8})	3.72×10^{-7} (2.6×10^{-8})	1.19×10^{-7} (4.2×10^{-9})	1.28×10^{-7} (1.3×10^{-9})	8.17×10^{-8} (8.2×10^{-11})

**$H^*(10)$ (μSv per primary) at Position 2**							
**Ion**	**LUPIN**	**WENDI II**	**LINUS**	**Pass. LINUS**	**BIOREM**	**MCNP**	**FLUKA**
**p**	1.29×10^{-10} (5.9×10^{-12})	1.28×10^{-10} (4.2×10^{-12})	1.18×10^{-10} (5.4×10^{-12})	1.00×10^{-10} (6.9×10^{-12})	1.18×10^{-10} (5.6×10^{-12})	9.08×10^{-11} (9.1×10^{-13})	9.24×10^{-11} (4.6×10^{-13})
**^{4}He**	9.73×10^{-10} (4.4×10^{-11})	1.03×10^{-9} (3.4×10^{-11})	9.09×10^{-10} (4.1×10^{-12})	8.83×10^{-10} (7.0×10^{-11})	7.26×10^{-10} (3.6×10^{-11})	9.22×10^{-10} (9.2×10^{-12})	8.70×10^{-10} (8.7×10^{-13})
**^{12}C**	6.22×10^{-9} (2.8×10^{-10})	6.19×10^{-9} (2.0×10^{-10})	5.66×10^{-9} (2.6×10^{-10})	5.46×10^{-9} (4.0×10^{-10})	5.74×10^{-9} (2.8×10^{-10})	4.86×10^{-9} (4.9×10^{-11})	3.72×10^{-9} (3.7×10^{-12})
**^{16}O**	1.03×10^{-8} (4.7×10^{-10})	1.06×10^{-8} (3.5×10^{-10})	9.43×10^{-9} (4.3×10^{-10})	9.01×10^{-9} (8.9×10^{-10})	9.28×10^{-9} (4.6×10^{-10})	6.17×10^{-9} (6.2×10^{-11})	5.37×10^{-9} (1.1×10^{-11})

**$H^*(10)$ (μSv per primary) at Position 3**							
**Ion**	**LUPIN**	**WENDI II**	**LINUS**	**Pass. LINUS**	**BIOREM**	**MCNP**	**FLUKA**
**p**	5.30×10^{-11} (2.5×10^{-12})	5.05×10^{-11} (1.7×10^{-12})	4.73×10^{-11} (2.2×10^{-12})	4.73×10^{-11} (4.3×10^{-12})	6.26×10^{-11} (2.8×10^{-12})	2.94×10^{-11} (2.9×10^{-13})	2.44×10^{-11} (2.0×10^{-13})
**^{4}He**	3.98×10^{-10} (1.8×10^{-11})	3.73×10^{-10} (1.2×10^{-11})	3.55×10^{-10} (1.6×10^{-11})	3.55×10^{-10} (6.0×10^{-11})	4.62×10^{-10} (2.0×10^{-11})	2.23×10^{-10} (2.2×10^{-12})	2.16×10^{-10} (6.5×10^{-12})
**^{12}C**	2.41×10^{-9} (1.1×10^{-10})	2.29×10^{-9} (7.5×10^{-11})	2.16×10^{-9} (9.8×10^{-11})	2.16×10^{-9} (2.3×10^{-10})	2.68×10^{-9} (1.3×10^{-10})	1.22×10^{-9} (1.2×10^{-11})	9.67×10^{-10} (3.7×10^{-12})
**^{16}O**	3.98×10^{-9} (1.8×10^{-10})	3.99×10^{-9} (1.3×10^{-10})	3.57×10^{-9} (1.64×10^{-10})	3.57×10^{-9} (5.31×10^{-10})	4.64×10^{-9} (2.7×10^{-10})	1.69×10^{-9} (1.7×10^{-11})	1.44×10^{-9} 4.3×10^{-12})

**$H^*(10)$ (μSv per primary) at Position 4**							
**Ion**	**LUPIN**	**WENDI II**	**LINUS**	**Pass. LINUS**	**BIOREM**	**MCNP**	**FLUKA**
**p**	6.18×10^{-11} (2.9×10^{-12})	6.15×10^{-11} (2.0×10^{-12})	5.98×10^{-11} (2.7×10^{-12})	7.09×10^{-11} (7.4×10^{-12})	7.51×10^{-11} (4.2×10^{-12})	6.22×10^{-11} (6.2×10^{-13})	4.28×10^{-11} (2.1×10^{-13})
**^{4}He**	3.64×10^{-10} (1.7×10^{-11})	3.55×10^{-10} (1.2×10^{-11})	3.25×10^{-10} (1.5×10^{-11})	3.20×10^{-10} (2.7×10^{-11})	4.42×10^{-10} (2.0×10^{-11})	3.08×10^{-10} (3.1×10^{-12})	2.92×10^{-10} (5.8×10^{-13})
**^{12}C**	1.66×10^{-9} (7.6×10^{-11})	1.66×10^{-9} (5.5×10^{-11})	1.63×10^{-9} (7.4×10^{-11})	1.55×10^{-9} (1.3×10^{-10})	2.08×10^{-9} (2.7×10^{-10})	1.28×10^{-9} (1.3×10^{-11})	1.05×10^{-9} (4.2×10^{-12})
**^{16}O**	2.63×10^{-9} (1.2×10^{-10})	2.72×10^{-9} (9.0×10^{-11})	2.47×10^{-9} (1.2×10^{-10})	1.98×10^{-9} (2.7×10^{-10})	3.27×10^{-9} (1.9×10^{-10})	1.68×10^{-9} (1.7×10^{-11})	1.49×10^{-9} (1.8×10^{-11})

Table S2: Ambient dose equivalent measured by the five rem counters and calculated via MC simulations in the four positions and for the four ions, normalised to unit therapeutic dose at the isocentre. The absolute uncertainties are given in brackets at one standard deviation.

$H^*(10)$ (µSv/Gy) at Position 1

Ion	LUPIN	WENDI II	LINUS	Pass. LINUS	BIOREM	MCNP	FLUKA
p	27.8 (1.3)	31.6 (1.0)	25.5 (1.6)	25.8 (1.9)	19.2 (1.12)	25 (0.3)	41 (0.1)
^{4}He	224.4 (10.2)	329.0 (10.8)	230.1 (10.4)	197.2 (13.7)	102.0 (5.5)	162 (1.6)	170 (0.1)
^{12}C	963.3 (43.6)	1367.5 (44.8)	812.1 (36.8)	823.9 (56.4)	358.0 (19.0)	443 (4.4)	226 (0.1)
^{16}O	1295.6 (58.7)	1870.0 (61.3)	1279.2 (57.9)	979.2 (67.2)	313.0 (17.9)	403 (4.0)	229 (0.2)

$H^*(10)$ (µSv/Gy) at Position 2

Ion	LUPIN	WENDI II	LINUS	Pass. LINUS	BIOREM	MCNP	FLUKA
p	9.24 (0.4)	9.18 (0.3)	8.5 (0.4)	7.2 (0.5)	8.4 (0.6)	7.37 (0.1)	7.34 (0.04)
^{4}He	19.17 (0.9)	20.20 (0.7)	17.9 (0.8)	17.4 (1.4)	14.3 (1.0)	20.1 (0.2)	18.3 (0.02)
^{12}C	24.80 (1.1)	24.70 (0.8)	22.6 (1.0)	21.8 (1.6)	22.9 (1.5)	25.6 (0.3)	16.0 (0.02)
^{16}O	26.98 (1.2)	28 (0.9)	24.8 (1.12)	23.7 (2.4)	24.4 (1.6)	19.3 (0.2)	15.2 (0.03)

$H^*(10)$ (µSv/Gy) at Position 3

Ion	LUPIN	WENDI II	LINUS	Pass. LINUS	BIOREM	MCNP	FLUKA
p	3.81 (0.2)	3.63 (0.1)	3.4 (0.2)	3.4 (0.3)	4.5 (0.3)	2.39 (0.02)	1.93 (0.02)
^{4}He	7.84 (0.4)	7.34 (0.2)	7.0 (0.3)	9.0 (1.2)	9.1 (0.6)	4.85 (0.05)	4.56 (0.01)
^{12}C	9.62 (0.4)	9.12 (0.3)	8.6 (0.4)	8.6 (1.0)	10.7 (0.7)	6.42 (0.06)	4.17 (0.02)
^{16}O	10.47 (0.5)	10.50 (0.4)	9.4 (0.4)	12.6 (1.4)	12.2 (0.9)	5.28 (0.05)	4.03 (0.01)

$H^*(10)$ (µSv/Gy) at Position 4

Ion	LUPIN	WENDI II	LINUS	Pass. LINUS	BIOREM	MCNP	FLUKA
p	4.44 (0.2)	4.42 (0.2)	4.3 (0.2)	5.1 (0.5)	5.4 (0.4)	5.05 (0.05)	3.40 (0.02)
^{4}He	7.18 (0.3)	7.00 (0.2)	6.4 (0.3)	6.3 (0.5)	8.7 (0.6)	6.69 (0.07)	6.15 (0.01)
^{12}C	6.61 (0.3)	6.62 (0.2)	6.5 (0.3)	6.2 (0.5)	8.3 (0.6)	6.74 (0.07)	4.51 (0.02)
^{16}O	6.93 (0.3)	7.15 (0.2)	6.5 (0.3)	5.2 (0.7)	8.6 (0.6)	5.25 (0.05)	4.16 (0.01)
